# A high performance hybrid LSTM CNN secure architecture for IoT environments using deep learning

**DOI:** 10.1038/s41598-025-94500-5

**Published:** 2025-03-20

**Authors:** Priyanshu Sinha, Dinesh Sahu, Shiv Prakash, Tiansheng Yang, Rajkumar Singh Rathore, Vivek Kumar Pandey

**Affiliations:** 1 Department of Electronics and Communication, University of Allahabad, Prayag Raj, Uttar Pradesh India; 2 SCSET, Bennett University, Plot Nos 8, 11, TechZone 2, Greater Noida, Uttar Pradesh 201310 India; 3 Pontypridd, University of South Wales, Newport, UK; 4 Cardiff School of Technologies, Cardiff Metropolitan University, Cardiff, UK

**Keywords:** IoT security, Deep learning, Hybrid LSTM-CNN, Intrusion detection, Cybersecurity, Threat detection, Machine learning, Computer science, Information technology

## Abstract

The growing use of IoT has brought enormous safety issues that constantly demand stronger hide from increasing risks of intrusions. This paper proposes an Advanced LSTM-CNN Secure Framework to optimize real-time intrusion detection in the IoT context. It adds LSTM layers, which allow for temporal dependencies to be learned, and CNN layers to decompose spatial features which makes this model efficient in identifying threats. It is important to note that the used BoT-IoT dataset involves various cyber attack typologies like DDoS, botnet, reconnaissance, and data exfiltration. These outcomes present that the proposed LSTM-CNN model has 99.87% accuracy, 99.89% precision, and 99.85% recall with a low false positive rate of 0.13% and exceeds CNN, RNN, Standard LSTM, BiLSTM, GRU deep learning models. In addition, the model has 90.2% accuracy in conditions of adversarial attack proving that the model is robust and can be used for practical purposes. Based on feature importance analysis using SHAP, the work finds that packet size, connection duration, and protocol type should be the possible indicators for threat detection. These outcomes suggest that the Hybrid LSTM-CNN model could be useful in improving the security of IoT devices to provide increased reliability with low false alarm rates.

## Introduction

**Figure 1:**
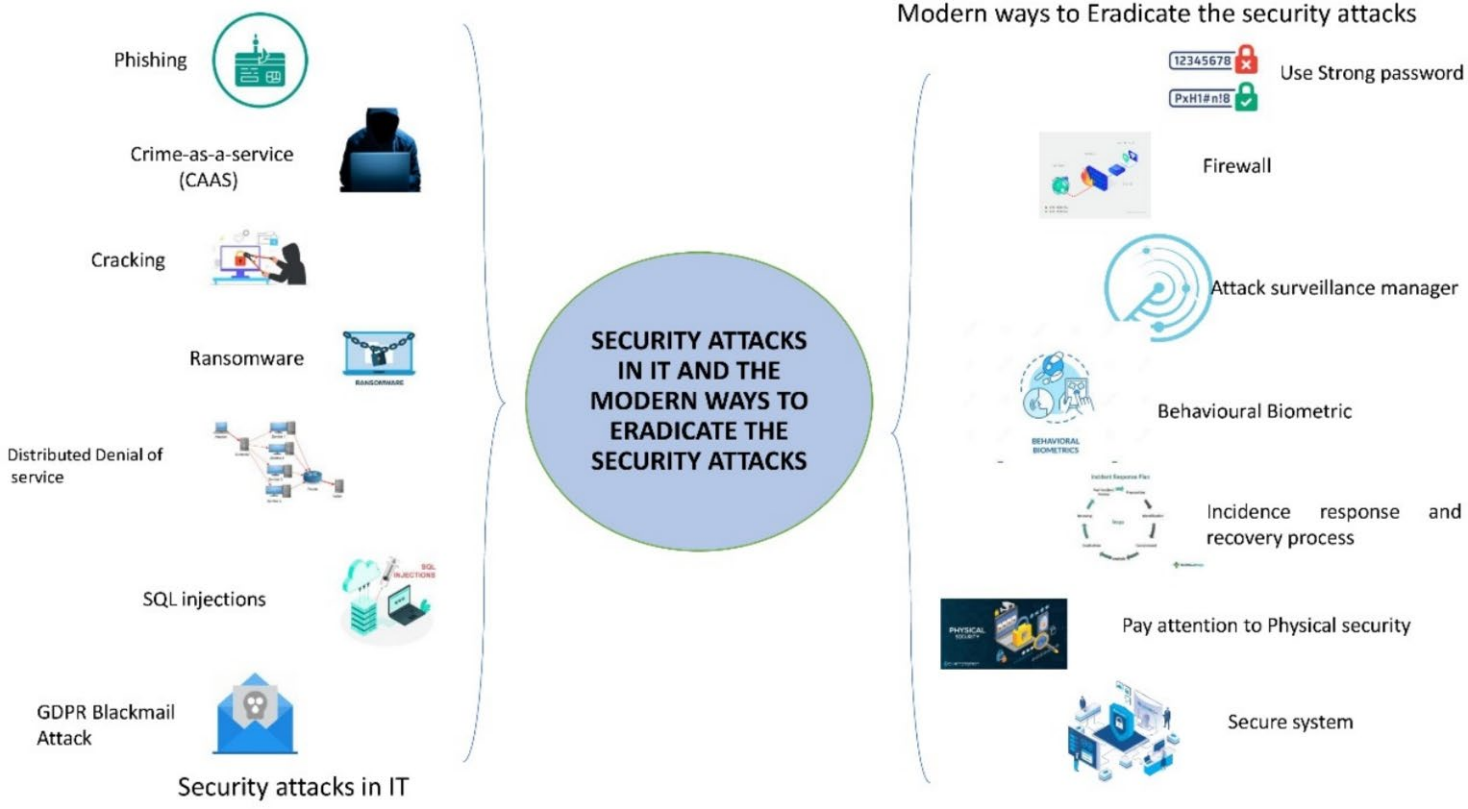
Security attacks in IT and the modern ways to eradicate the security attacks.

People become more and more dependent on technologies and their progress in modern society. The utilization of scientific knowledge for practical applications or purposes in industry or in daily life is termed technology. The innovations developed in the 19th century have been altered. In the 21st century, the advancement of technology has enhanced more. IT is developed owing to the advancement of the technology^1,2^. The term IT is more comprehensive. All types of technology that operate in the automation of an industrial process, information systems, or even in the personal utilization of computational resources are referred to as IT. A set of related fields that comprise computer systems, information processing, software, programming languages, and storage is also termed IT, which involves information processing and incorporates communication via electronic equipment^3,4^. Innovation and development via advancements in AI, cyber security, CC, IoT, software, analytics, computers, networking, Application Programming Interfaces (APIs), audio-visual technologies, databases and the Internet, and Governance are promoted by IT. These ITs provide benefits by enhancing communication, increasing operational efficiency, and improving the quality of decision-making and driving innovation, thus increasing the efficiency and productivity of secured information^5^. Information is becoming one of the most valuable assets of any state or organization. It is also becoming increasingly valued and guarded. Since the current generation depends upon the information system, the issue that happens in the information system is either caused by computer viruses, threats, or abuse. Hacking, spamming, sniffing, viruses and malicious software, jamming, and identity theft are the current problems that happen in IT^6,7^. Simultaneously, this information is exposed to several threats, namely cyber-attacks and breaches of data privacy. Hence, information security has become one of the most significant problems^8,9^. Due to improper planning for security in the advanced technology, this information security issue happens. Furthermore, the chance of risk in IT development is created by weak security. The figure 1 describes the security attacks in IT and the modern ways to eradicate the security attacks.

CC, AI, cyber security, and IoT are some of the significant ITs affected by security issues. The security issues in these technologies are described in the following paragraph

1. Cloud computing: One of the biggest concerns affecting the growth of CC is security issues, which add complications, such as data loss, insecure APIs, interference of hackers, and user account hijacking^10^.
2. AI: Adversarial ML, data poisoning, data privacy, bias and discrimination, supply chain attacks, model theft, network security, vulnerability, and automated malware generation are some of the security issues that happen in AI.
3. Cyber security: Some security issues faced in cyber security are Distributed Denial of Service (DDoS) attacks and Intrusion Detection and Prevention System (IDPS) that generate large numbers of false alarms, thereby distracting cyber security experts from finding real threats^11,12^.
4. IoT: IoT networks are prone to several security attacks, namely weak passcodes, insecure passwords, information theft and unknown exposure, out-dated software, insufficient testing and updating, malware, prioritized Wi-Fi security, regular checks for patches and updates, secure, and unencrypted data transmissions^13,14^.

**Figure 2:**
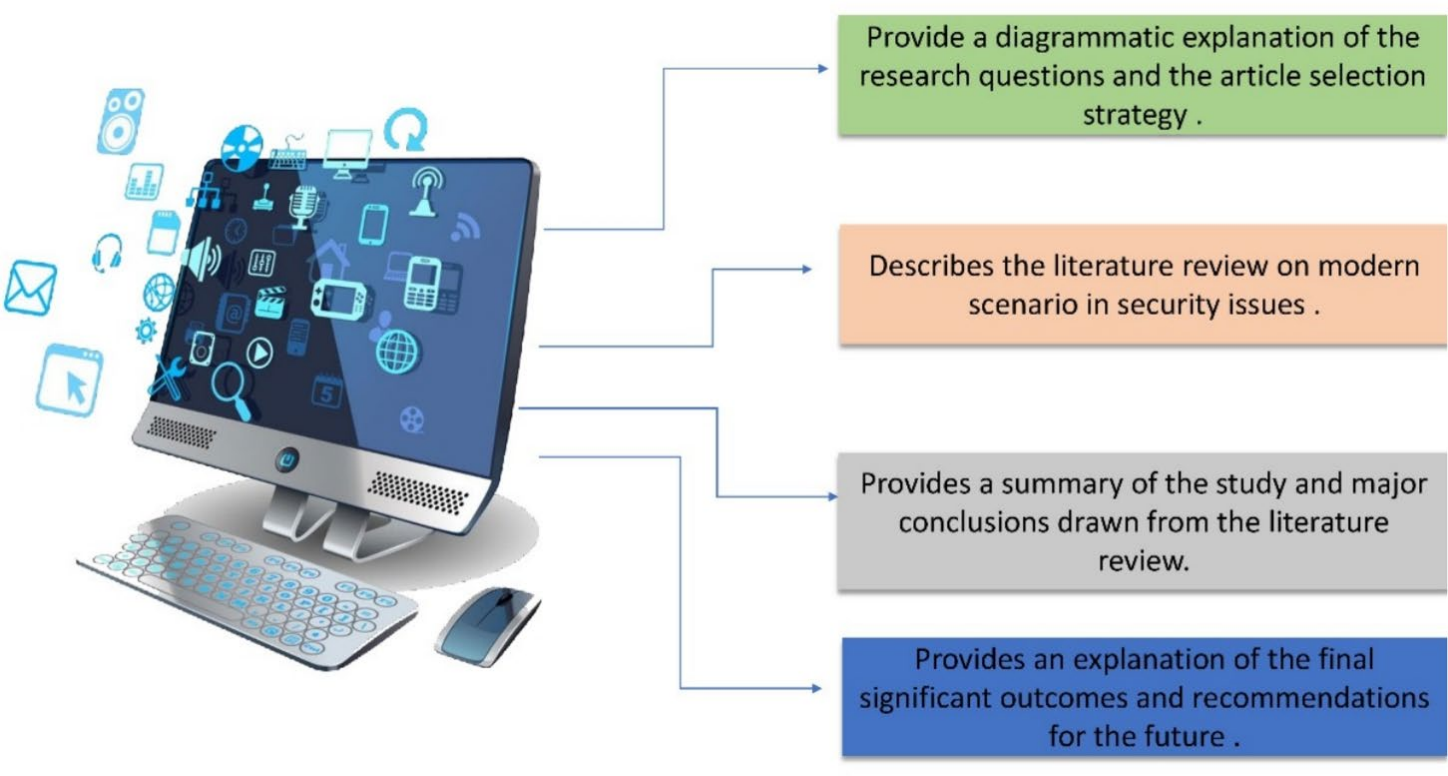
The system review scenario for IoT Environment.

To overcome security issues in CC, AI, cyber security, and IoT, certain efficient and modern ways are available. Some of the criteria, such as utilizing strong passwords, AI video surveillance, firewalls, attack surface management, cloud security, behavioral biometrics, conducting penetration testing, enabling two-factor authentication, IoT security, encryption, regulation, threat intelligence, and strong authentication can protect these ITs^15,16^. These criteria aid IT effectively; they also maximize the productivity and effectiveness of the systems, resources, and processes. Hence, a sustainable solution for effective IT is provided by modern improvement^17^. After the completion of the introduction section 1, The figure 2 explains the remaining sections of this paper in brief.

### Research objectives

The research questions include the following:

1. To propose a hybrid LSTM-CNN model for intrusion detection in IoT settings through the application of deep learning.
2. In an effort to improve the accuracy of identifying cybersecurity threats, temporal (LSTM) and spatial (CNN) features extraction techniques are proposed.
3. To test the proposed model against adversarial examples and other models that serve as baselines: CNN, RNN, BiLSTM, and GRU.
4. To develop new and highly efficient algorithms for intrusion detection systems (IDS) in order to minimize false positive results while increasing the rates of detection.
5. To evaluate the proposed model, the BoT-IoT dataset containing real-world cyber threats like DDoS, botnets, reconnaissance, and data exfiltration attacks needs to be used.

### Key contributions

This study has the following research contributions:

1. Proposes an Enhanced Hybrid LSTM-CNN architecture that integrates LSTM layers that consider temporal dependencies while CNN layers to capture spatial feature and results in enhanced and precise detection of intrusions.
2. It incorporates an enhanced mechanism of anomaly detection that has a much lower false positive rate of approximately 0.13% which improves on the reliability of the IoT security framework.
3. Assesses how much the model is resistant to attacks, proves that LSTM-CNN achieves 90.2% accuracy under worst case scenario while CNN is rated 82.0
4. Reduces the computational load and improves its efficiency to provide an inference time of 2.3 milliseconds per sample to support real-time intrusion detection on IoT systems.
5. Categorizes into basic CNN, simple RNN, standard LSTM, BiLSTM, and GRU and establishes a benchmark among them.

While many approaches involve the use of deep learning methodologies such as CNNs, LSTMs, and hybrid models, the current work was designed to combine the promising techniques in a novel and optimized way for IoT security. In contrast to the CNN model or LSTM model, our Hybrid LSTM-CNN model employs both space and time, which make it easier for the model to identify cyber threats accurately.

The organization of the rest of this paper is as follows: Section 2 provides a general insight into the objectives and limitations of existing intrusion detection mechanisms in general and more specifically, the machine learning based, and deep learning based methods. In section 3, the hybrid LSTM-CNN is presented with descriptions about its structures, mathematical equations to evaluate security risks. The hardware and software environments are described in section 4 and the subject of this section is the preparation of datasets and the process of training and testing the model. In section 5, assessment measures of the proposed model including accuracy, precision, recall, F1-score, false positive rate, and efficiency are shown and further comparison is made with the baseline models. Lastly, Section 6 presents the overall conclusion of the paper, research contributions, and potential future works that can be developed from this study such as federated learning to reduce the amount of data transfer while collecting new IDS models and data from IoT devices, reinforcement learning to design IDS for constantly changing IoT networks, and incorporating the blockchain technology into the proposed IDS models to enhance security in IoT networks.

## Related work

One of the first methodological steps in undertaking research is Research Questions (RQs). In this article, the RQs are described grounded on the following categories:

1. In this article, the RQs efficiently guided the research project.
2. An effective direction for the collection of suitable data was provided by RQs,which also aided in the analyses of the data.

**Figure 3:**
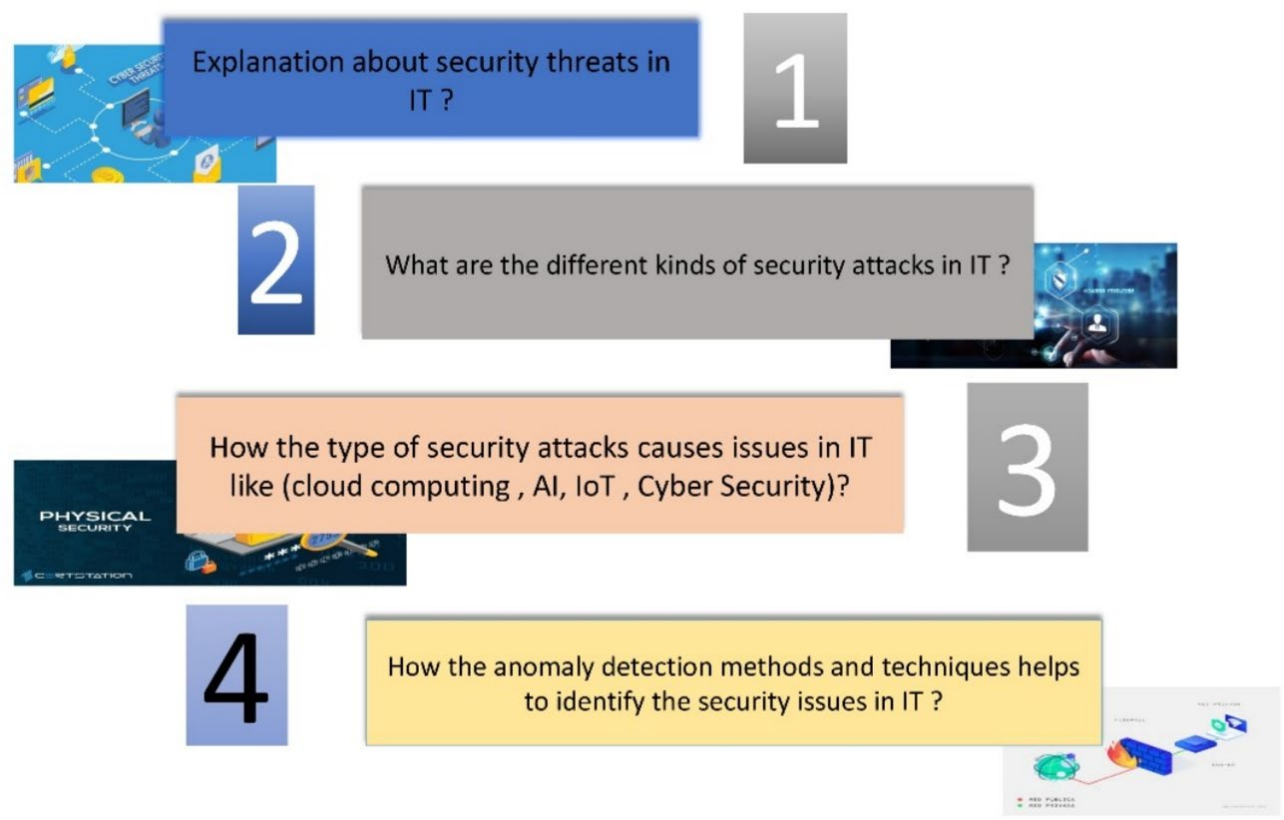
The Research Questions for Systematic Review in IoT Environment.

The RQs are framed centered on the given title and the focused areas of the objectives. These are classified into four types, which are depicted in the following sections. Grounded on this, The figure 3 describes the classification of the RQs.

The process of selecting the suitable type for review is termed article selection. The overall outcome and quality of the work are significantly influenced by the article selection process. Diverse types of article selection in this research have a unique identity. Mainly, 2 types of processes were involved in eligibility criteria. The processes were inclusion and exclusion criteria. In this process,the type of studies that were added and removed was decided in an efficient way. The included parts of this are,

- The related articles that stated the security problems in IT were included.
- Articles reporting on Anomaly Detection (AD) methodologies and techniques in IT for security issues were included.
- Articles that were published betwixt 2015 and 2024 were included.

The excluded parts are as follows,

- The articles that reported on network and communication technology security problems were excluded.
- In this study, the article other than IT was excluded.
- The articles published before 2015 were excluded.

The related work section focused on research materials associated with the modern scenario for security issues within the time frame from 2015 to 2024.

Databases: To recognize the papers, important databases, encompassing Science Citation Index Expanded (SCIE), Scopus, and Web of Science (WOS), were utilized.

Database Insights: Emerlad, Oxford University Press, and IGI Global Publishing were found to be distinct from other significant databases. In Emerald, Oxford University Press, and IGI Global Publishing, scientific journals and conference proceedings were available. Therefore, they were the best resource for researchers.

**Figure 4:**
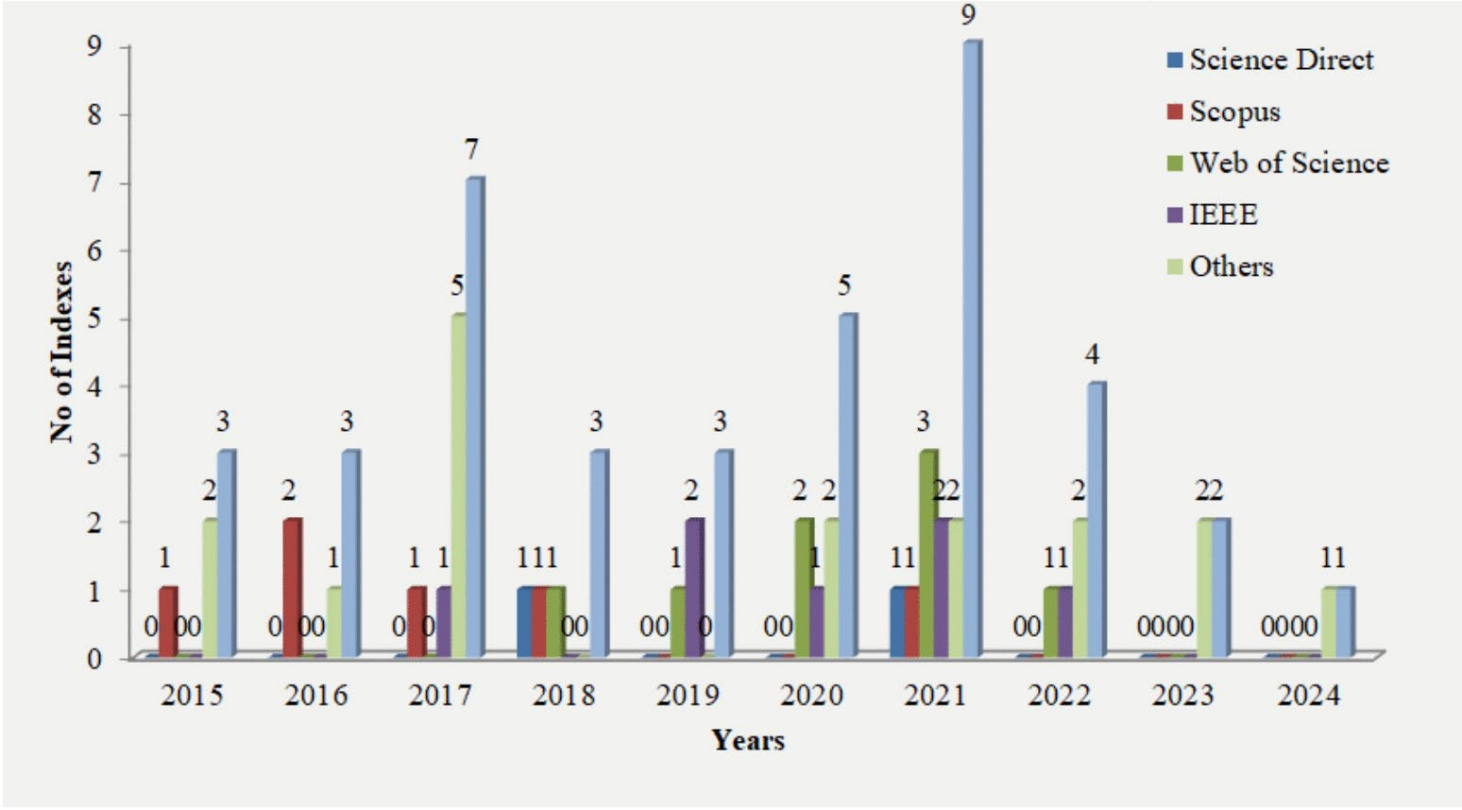
Distribution of different databases of scientific literature.

Paper selection: For this related work section, 40 papers were chosen. The papers were selected and recognized centered on the given criteria. After investigating the search details, The figure 4 briefly explains the search outcome of this literature survey.

A malicious act that intends to steal or corrupt data or disrupt the entire system is termed a security threat. This security event refers to an occurrence, where data or its network may have been exposed. Malware, phishing, DDoS attacks, ransomware, insider threats, social engineering, viruses, IoT security, man-in-the-middle attacks, passwords, advanced persistent threats, along with injection attacks are some security issues developed in the technology. In this review, the modern scenario for the reduction of security problems in the IT system centered on the assessment process of how security threats happen in IT by analyzing the diverse security attacks and how they occur in IT is described. Furthermore, the brief review is grounded on the diverse types of security attacks in IT and how they happen in CC, cyber security, AI, and IoT. The AD techniques in IT to reduce the problems associated with security issues are also explained in this review.

### Security threats in IT

An event or occurrence that negatively impacts IT is termed a security threat, which can damage core processes and the mission^18,19^. This general security risk happens when sensitive information is without awareness or permission^20^. In IT, CC plays a vital role in data backup^21^. A network of physical devices that can transfer data to one another without human intervention is referred to as IoT. Computer systems are protected from scams and data theft by cyber security^22,23^. This section describes the articles associated with IoT, cyber security, CC, and their security issues like Phishing Attacks (PAs), DDoS, and drive-by download attacks. Syed et al.^24^ described how to recognize and reduce PA threats in IoT utilizing a threat modeling framework. The threat-modelling approach was utilized for identifying and diminishing potential threats that caused a PA. The outcomes stated that the potential threats in IoT devices as well as systems were recognized in the early design stages for ensuring the IoT devices’ secure deployment in critical infrastructures. The possibility of perpetrating PAs in IoT contexts was still limited. Mohammed et al.^25,26^ described the threat investigation and DDoS attack recognition in the IoT. For the threat analysis and attack recognition in IoT, the intrusion detection method was utilized. According to the results, this study obtained a detection rate of 98.98% and an accuracy of 99.29% with minimal processing complexity. This study also attained a performance ratio of 90.26%. Computation complexity was higher in this study. Thomas &Houssain^27^ presented the botnets’ security threats to cyber systems. The shared malicious packet characteristics were investigated by the signature-centric detection method with known botnet traffic signatures. The results depicted that the applied method attained accurate efficiency in the test environments. Mobile botnet devices generally suffered from less battery power and limited bandwidth. Kenneth et al.^28^ explained valuing information security from a PA. To assess information security from a PA, this study utilized a trade-off elicitation methodology. According to the investigation, the outcomes offered valuable insights for the design of more usable information security systems. For the assessment of the results, limited real-world data was available^29,30^. Ibrahim & Vaclav^31^ explained the malicious file hash detection as well as drive-by download attacks. To detect any malicious file downloaded by the network hosts, the intrusion detection system was utilized. The analysis showed that for every single day, the malicious file hashes’ blacklist was automatically updated. Moreover, the detection was in real-time. In this research, the detection methodology was only centered on malicious file hashes’ blacklist^32,33^.

### Different types of security attack in IT

A kind of malicious attack that happens in IT is a security attack. Any kind of malicious activity that occurs in IT attempts to gather, deny, disrupt, destroy, or degrade information system resources or the information itself^34,35^. In some IT sectors, namely CC, cyber security, IoT, and AI, security attacks can happen^36^. DDoS, Phishing, Structured Query Language (SQL) injection, ransomware, password attacks, Domain Name System (DNS) tunneling, drive-by attacks, insider threats, malware attacks, zero-day exploits, spoofing, session hijacking, botnets, eavesdropping, and Trojan are some of the diverse types of attacks that occur in IT^37^. Table 1 describes the articles associated with the types of security attacks in IT.

**Table 1:** Summary of Authors, Objectives, Security Attacks, Outcomes, and Limitations.

Authors’ names	Objectives	Types of security attacks	Outcomes	Limitations
Okusi & Oluwatobiloba,^38^	To utilize the AI-based techniques Deep Forest (DF) model for addressing the problem of class imbalance in some cybersecurity systems.	Cross-Site Scripting Attacks	As per the analysis results, the applied DF model aided in addressing the issue of class imbalance, which was neglected by extant studies.	In this study, the AI technologies utilized were intensive, time-consuming, and required a training dataset.
Annie,^39^	To recognize the several security problems arising from using cloud services, particularly hijacking accounts/services of CC.	Account Hijacking	The analysis depicted that the hacker’s data breach of a network administrator’s login credentials could cause access to an entire network.	It might take a year to crack an encryption key owing to hardware drawbacks.
Hawzhin, et al.^40^	To use the Power Profiling (PP) as well as Network Traffic (NT) data without intervening in the Integrated Circuits (ICs) design for detecting malicious activity in the Home Area Network (HAN).	Trojan Attacks	According to the results, all the attacks could be concurrently detected by the applied technique with an accuracy of 92%.	The Internet of Things-Edge Devices (IoT-EDs) had a limited power budget. Hence, the power consumption was increased by the extra computation requirement.
Tri & Myungsik,^41^	To utilize software-defined networking and IoT for enhancing the management and control capabilities of IoT networks.	Eavesdropping attack	The outcomes depicted that since the applied hybrid countermeasure could prevent all link spoofing attacks in real-time, it was feasible and effective.	When contrasted with the Transmission Control Protocol (TCP) bridge, the TCP dump and TCP replay took longer processing time.
James, et al.^42^	To detect the man-in-the-middle attacks with minimal intervention as well as workload to the network and systems centred on the trusted time server.	Man-in-the-middle attack	According to the investigation, the trusted time server would mark the message as suspicious and send essential notifications to the users.	Nevertheless, employing a time server was complicated, with various practical limitations.

### Anamoly detection method and techniques in IT for security issues

Business, IT, and application performance can be enhanced by AD systems^43^. The detection of fraud, security incidents, and opportunities for innovation can also be improved by these AD systems. Suspicious activity, which falls outside the established standard patterns of behavior, is recognized by AD^44^. Genetic Algorithm (GA), artificial immune system, and Differential Evolution (DE) are some AD techniques utilized in this study centered on the evolutionary methods. Techniques, namely entropy and Kullback-Leibler distance are used in the information theory methods. To resolve security issues, the statistical method uses principal component analysis, covariance matrix, and wavelet techniques. Table 2 describes the articles associated with the AD methods and techniques in IT for security issues.

**Table 2:** Summary of Anomaly Detection Techniques, Methods, Results, and Limitations.

Authors’ name	Objectives	Anomaly detection techniques and methods	IT domain	Results	Limitations
Anderson, et al.^45,46^	To utilize the GA for generating the network segment’s digital signature to predict the NT behavior for a given time interval.	GA (Evolutionary methods)	Computer networks	Accuracy: 96.53%, False positive rate: 0.56%	Network administrators had limited resources and time for analyzing whether a real threat was signified by an alarm.
Sahar, et al.^47^	For classifying IoT intrusion and minimizing the generation of false alarms.	Artificial Immune System (Evolutionary methods)	IoT	Accuracy: 98.73%	Most of the devices were expensive and small. Also, they had limited memory and calculating capacity for running the security software.
Ebenezer Aderemi,^48^	To find the optimal feature set utilizing DE from standard intrusion dataset.	DE (Evolutionary methods)	AI	Accuracy: 81.59%	To discrete optimization, the applied DE was limited.
Ajay, Rohit,^49^	For recognizing the entropy-based AD to improve the network security.	Entropy (Information theory methods)	CC	According to the outcomes, the except threshold value was ($\beta$) = 0.1.	The data gathered was limited to the selection criteria.
Qirui, et al.^50^	To analyze the design of online stealthy attacks for moving the system’s state to a desired target.	Kullback-Leibler distance (Information theory methods)	Cyber security	The analysis stated that the convex optimization issue occurred to calculate the attack’s mean and covariance.	A much higher computational cost was required for the analytical expression of the optimal stealthy attack.
Yusuf et al.^51^	To utilize the edge-centric blockchain-enabled AD for recognizing insider attacks in IoT.	Edge-centric Blockchain-Enabled AD	IoT	Assessment of the technique utilizing real IoT system datasets depicted that the intended purpose was attained by the applied technique when ensuring the data’s integrity and availability.	For the deployment of IoT systems, the availability of the data was critical.
Manimurugan,^52^	This study utilized the incorporated architecture of the Internet of Things-Fog-Cloud Computing (IoT-Fog-CC) model to investigate security attacks.	Principal component analysis (Statistical-based method)	CC	Accuracy: 92.48%, Detection rate: 95.35%	The applied technique failed to solve the false positive rate issues of the AD system.
Poovendran,^53^	To detect the Distributed Denial-of-Service-Hypertext Transfer Protocol (DDoS-HTTP) attacks in cloud environments grounded on the covariance matrix technique.	Covariance matrix (Statistical-based method)	CC	Accuracy rate: 92%, Low false positive rate: 3%	Complicated or unique attack strategies were not supported by the covariance matrix technique.

**Figure 5:**
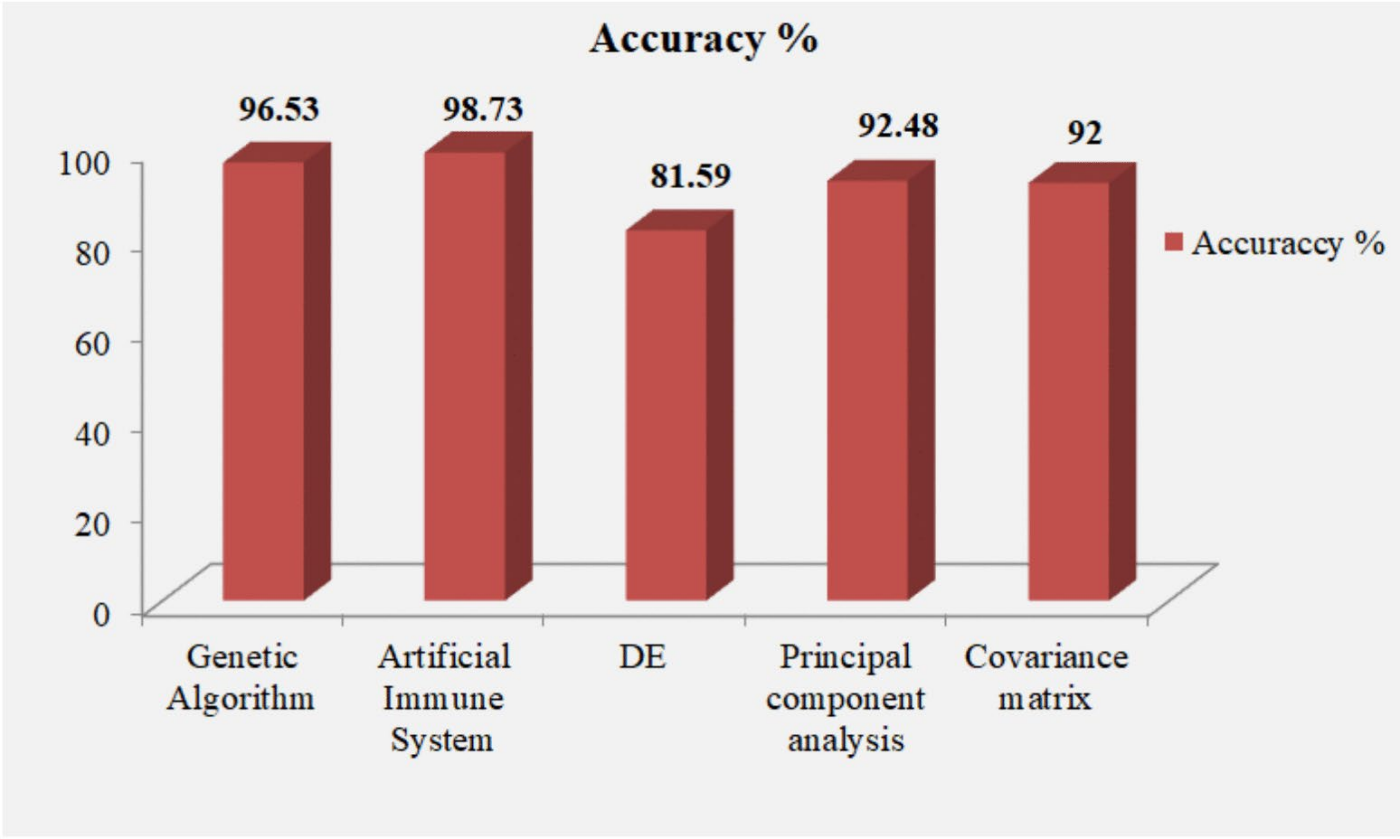
Accuracy values along with the techniques of anomaly detection in IT.

GA is one of the AD techniques that provide a better accuracy rate for security-related problems. Anderson et al.^46^ described the explanation centered on GA in AD. Sahar et al.^47^ described other techniques like artificial immune system^47^ based analysis in IoT. Ebenezer &Aderemi^47^ explained the DE^48^ analysis techniques utilized in recognizing the security issues in AI. Lastly, the principal component analysis-centric techniques utilized in CC was described by Poovendran^53^. The figure 5 explains some of the accuracy values and the AD techniques in IT.

Hence, it was found from the analysis that the artificial immune system-based evolutionary method obtained the highest accuracy of 98.73%, which was investigated by Sahar et al.^47^. Unknown attacks are efficiently detected by Artificial immune-centric intrusion detection. To generate detectors, the evolution algorithm creates possible unknown attacks. The artificial immune systems’ field is concerned with the function of the immune system for computational purposes. This analyzes the application of these systems towards solving computational issues that happen owing to security associated with IT. John, et al.^54^ described the GA-centric feature selection and naive Bayes for AD in a fog computing environment. For diminishing the time complexity in the National Security Laboratory Knowledge Discovery Dataset (NSL-KDD), GA was used in this study. The outcomes displayed that the GA attained an overall performance rate of 99.73% accuracy with a false positive rate as low as 0.6%. Nevertheless, building a general attacks database as well as properly setting detection thresholds utilizing the dataset only happened in the training phase. David et al.^55^ explained AD in cloud platforms utilizing a wavelet-based framework. To detect anomalous behavior, the wavelet-centricAD framework was utilized for simultaneously investigating multiple monitored metrics. According to the investigation, the confidence interval was up to 99%. The given confidence interval was one of the drawbacks of this statistical method. It only recognized the points as anomalous (false positives). Stefano, et al.^56^ described the enhancement of the covariance-centric methodologies for statistical AD algorithms. To build a regular network traffic’s norm profile and detect anomalous activities in the data flow, this covariance matrix technique was utilized. The investigation depicted that concerning Receiver Operating Characteristic (ROC) curves, the applied algorithm’s performance was assessed and analyzed^57^

Former IDS models employed the signature based and rule based models, which are not helpful in the contemporary computer world full of emerging threats. To overcome this, deep learning such as CNN and LSTM have been employ for the purpose. CNN-based IDS models^58,59^ are good in capturing spatial features of the network traffic but lack the capability to model temporal dependencies therefore they are limited in handling complex cyber aggression. As for the second type, the use of LSTM as a sequential model to capture the temporal dependency enhances the attack classification accuracy^60,61^ at the cost of computational intensity. Thus, the modification of LSTM can be achieved through GRU models, which represent a more computationally efficient position and do not lose the ability to extract temporal characteristics^62^.There are various works done in recent past that attempts at using multiple forms of deep learning in order to improve detection accuracy. For instance, works as^63,64^ proposed different approaches based on CNN-LSTM models and while proving better results also noted the problem of overfitting and high false positive rate^65^. However, the existing approaches to combining the classifiers have a problem of being sensitive to adversarial attacks^66^ and are not easily understandable when it comes to the decision making process. To the best of our knowledge, our proposed Enhanced Hybrid LSTM-CNN model is the first that incorporates both spatial and temporal feature extraction towards enhancing the intrusion detection rates^67^ with low false alarms. Our work differs from regular LSTM models since feature selection in this work utilized SHAP-based feature importance analysis which makes it more understandable as well. Besides those, we conduct robustness testing, and the result shows that our model still has 90.2% accuracy even under the adversarial attack circumstance, which is an important aspect often neglected by others. Moreover, the careful selection of hyperparameters and methods minimizing overfitting such as dropout, L2 regulation, and early stoppage are the key differences between our work from the other similar studies.As such, the implemented enhancements enrich our study and help to advance IDS solutions based on deep learning for IoT networks, which had the aforementioned limitations.

### Research gap

Although IDS for IoT has received much attention in recent years, recent studies in IDS for IoT are still lacking in certain ways, including accuracy and adaptability as well as robustness against attacks. Two well-known IDS models are the rule-based and the anomaly-based IDS: There remain some disadvantages of traditional IDS: there is a problem of high false positive rate, long time of computation, and in means of not recognizing new and distinct forms of attacks. Previous studies of the intrusion detection applying machine learning approach revealed enhancement in the classification results, but failed to address feature extraction problem and limited representation of temporal-spatial characteristics of network traffic. The CNN, RNN, LSTM and GRU based models have comparatively good detection performance but they do not incorporate temporal structure analysis with spatial pattern extraction effectively and efficiently and hence does not contribute to optimal detection of intrusions.

Additionally, previous work mainly concentrates on accuracy enhancement; however, the robustness of models to the adversarial attack has not been covered according to an experimental perspective, which is essential in real-world scenarios. Most of the proposed models lack a good trade-off between detection accuracy and complexity, thus cannot be used in real-world IoT applications. Besides, feature importance analysis is profound, yet few research has been conducted on the SHAP-based feature analysis to justify the identification of more network characteristics leading to threat detection. Although, a lot of progress has been made in using deep learning as a solution for IDS on IoT, yet the current techniques have the following drawbacks. Most Convolutional Neural Network-based IDS models are good at identifying the spatial characteristics of the network traffic patterns but weak in handling temporal relations, whereas LSTM-based IDS models are particularly good at capturing temporal characteristics of the traffic while they are not so adequate in capturing spatial characteristics of the traffic. Several studies have attempted to increase the detection accuracy using Hybrid CNN-LSTM, but the Glossary results yielded low interpretability because the approaches did not employ Explainable AI such as feature importance analysis.

Also, in the prior research, the adversarial robustness aspect is not very much discussed, which makes the IDS models sensitive to adversarial evasion attacks. In addition, several IDS models are developed with no consideration of class imbalance and this makes it difficult to predict as it always favours majority class attacks and has a tendency to misclassify the stealthy minority class intrusions. It fulfils these gaps through the augmentation of such features as SHAP-based feature selection for interpretability, SMOTE-based class balancing for fairness and adversarial robustness assessment.

To further illustrate the limitations of previous IDS models, Therefore, Table  3 shows a comparative summary of works related to the proposed methods. The table shows the differences concerning the feature extraction approach, class imbalance management, model interpretability, adversarial evaluation, and computational cost.

**Table 3:** Comparison of Existing IDS Models with the Proposed Hybrid LSTM-CNN Approach.

Study	Feature extraction	Handles class imbalance	Explainability (XAI)	Adversarial robustness	Computational efficiency
CNN-Based IDS	Spatial (CNN)	No	No	No	Moderate
LSTM-Based IDS	Temporal (LSTM)	No	No	No	High
Hybrid CNN-LSTM	Spatial + Temporal	No	No	No	High
XAIEnsembleTL-IoV	Transfer Learning + Explainability	No	Yes	No	Moderate
ResNet50-1D-CNN	Deep Transfer Learning	No	No	No	High
Proposed Hybrid LSTM-CNN	Spatial + Temporal	Yes (SMOTE)	Yes (SHAP)	Yes	Optimized

From table  3, it is evident that most of the existing IDS models have not incorporated class imbalance, adversarial robustness or explainability issues. Our Hybrid LSTM-CNN model incorporates CNN for the spatial feature extraction, LSTM for sequential learning, SHAP for the selection of the features, which can be easily explained, and SMOTE for handling the class imbalance problem when compared to the traditional models.

To fill these gaps, this paper presents the Enhanced Hybrid LSTM-CNN Secure Architecture that employs the LSTM for capturing long-term temporal patterns as well as CNN for spatial features to enhance intrusion detection performance. Unlike other models that have been developed earlier, this model is tested for adversarial attacks and its functionality is very strong not to be impacted negatively should there be an attack. Furthermore, it takes 2.3 ms per sample in terms of inference time which makes it possible to be implemented for real-time Security of IoT devices. On the information provide explanations about the most important features within the network traffic for threat detection to resolve the absence of explainability in previous IDS systems through the usage of the SHAP-based feature importance analysis. Therefore, this work propose a solution that is more practicable and safer for the current IoT scenarios by reducing the time and power consumption, with keeping a high level of accuracy in intrusion detection, providing adversarial robustness, and increasing the model interpretability.

## Proposed framework and mathematical model for addressing security threats in IT

Security threats in the IT systems are dangerous as they compromise the security of data as well as harm the network, hardware, and the cloud. The above threats can be categorized into four main categories: data threats that include data theft, SQL injection, and ransomware, network threats, which consist of DDoS, MITM, and spoofing, hardware threats that include the firmware and supply chain attack and the last being the cloud threats comprising APIs vulnerability and insecurity of the authentication mechanisms. To categorize these threats systematically, evidently, the set of security threats is defined as $T = \{T_1, T_2, ..., T_n\}$, where each $T_i$ emphasizes one specific type of security threat towards IT systems. In cybersecurity risk assessment, it is important that you have an understanding what are the levels of occurrence of certain threats. Historical attack data and newly appeared vulnerabilities determine the probability associated with each threat. In order to quantify this, we define the probability of occurrence for a given threat $T_i$ based on the frequency of observed attacks. A time-weighted probability model would improve accuracy by prioritizing recent attack data:

1 $$\begin{aligned} P(T_i, t) = \frac{w_t A_i}{\sum _{j=1}^{n} w_t A_j} \end{aligned}$$

where $w_t$ is a time decay function:

2 $$\begin{aligned} w_t = e^{-\lambda (T_{\text {current}} - T_i)} \end{aligned}$$

The decay factor $\lambda$ is included where it gives more weight to recent attacks. This prevents attack probabilities from being an aggregate of historical security trends instead. This provides that threats with more occurrences contribute more to the overall security risk assessment and hence allow organizations to better expend resources towards mitigation.

To evaluate the probability of success in an IT system ($P(A)$), the likelihood of successfully exploiting vulnerabilities is determined. It is given by the equation:

3 $$\begin{aligned} P(A) = 1 - \prod _{i=1}^{n} (1 - P_m(T_i))^{V_i} \end{aligned}$$

where $P_m(T_i)$ stands for the probability of gaining advantage from using vulnerability $T_i$ and $V_i$ is the number of exploited vulnerabilities in attack in $T_i$. This use of the product term refers to the multiple avenues through which the attack is done.These models better capture dependencies between attack types, making the system more robust to chained cyber threats. When threats are individual, then $P(A)$ is described by the following equation:

4 $$\begin{aligned} P(A) = 1 - e^{-\sum _{i=1}^{n} P_m(T_i)} \end{aligned}$$

which gives a more real-life representation to attack vectors, as the likelihood of each of them are estimated independently. However, when threats are correlated, as in the case of ransomware after phishing email has been sent, a Bayesian model is used to update the probability. For the given problem, the conditional probability, P of attack $A$ following a prior attack $B$, of an attack can be calculated using the subsequent formula.

5 $$\begin{aligned} P(A|B) = \frac{P(B|A) P(A)}{P(B)} \end{aligned}$$

where $P(A|B)$ is a conditional probability, the probability of an attack occurred and another attack also occurred, $P(B|A)$ is also a conditional probability the probability of attack $B$ given that $A$ has already occurred whereas $P(A)$ and $P(B)$ are prior probabilities of two attacks respectively. This helps to have a more realistic risk evaluation in information technology security especially when it comes to inter-connected or sequenced threats.

Security threat assessment How each threat $T_i$ impacts the three key security attributes namely; Confidentiality $C$, Integrity $I$ and Availability $A$. In order to express this in numerate terms we introduced a Security Impact Function $I(T)$, which aggregates these three predicates with weights.

6 $$\begin{aligned} I(T) = (W_c \cdot C + W_i \cdot I + W_a \cdot A)\times f(T) \end{aligned}$$

where $W_c, W_i, W_a$ are the weights obtained for each security attribute showing what proportion of the overall consideration each attribute holds, and $C, I, A$ are the impact values ranging from 0 to 1. These values assist in determining the extent to which a certain threat is likely to impact the system security. where $f(t)$ is a time-dependent severity function:

7 $$\begin{aligned} f(t) = 1 + \alpha \log (1 + T_{\text {recency}}) \end{aligned}$$

where $T_{\text {recency }}$ is the number of days since the attack was last seen and $\alpha$ is a scaling factor that increases the impact score depending on recency so that newer threatening attacks are prioritized very high.

Besides, the expected security risk $R$ is defined as:

8 $$\begin{aligned} R = \sum _{i=1}^{n} P(T_i) \cdot I(T_i) \end{aligned}$$

where $P(T_i)$ is the estimated probability of the threat $T_i$ to occur and $I(T_i)$ is the respective security impact of the threat. This formula yields an overall risk rating of the threats to a firm’s IT systems by summing the individual ratings on probability and impact, which gives a good way to measure and address a range of possible risks faced in system security.

Security risk management focuses on reducing the impact of risks and the cost of this reduction. This objective is expressed as:

9 $$\begin{aligned} \min _{x} \sum _{i=1}^{n} P(T_i) \cdot I(T_i) - \sum _{j=1}^{m} C_j x_jE_j \end{aligned}$$

The first of these is the summation of all the potential threats whereby the total risks that are incurred by firm are the of the probability of the threats $P(T_i)$ and the impact $I(T_i)$ that is incurred by the firm and $E_j$ represent the effectiveness of security measure j. The problem is to be limited by the financial resource which is in a certain situations constricted by budget limitations.

10 $$\begin{aligned} \sum _{j=1}^{m} x_j \le B \end{aligned}$$

where, $x_j$ is equal to 1 if the security measure $j$ is employed, otherwise it is equal to 0, $C_j$ is the cost of the security measure $j$, and $B$ is the total budget dedicated to security. With proper identification of the security measures to be put in place, it is possible to improve the security of the organization’s information technology gadgets while observing the costs.

It was ascertained that certain security measures rely on others that are to be in place, this means that if a particular security measure is needed then another one must also be in place. This dependency constraint is formulation as follows:

11 $$\begin{aligned} x_j \le x_k, \quad \text {if security measure } j \text { depends on } k \end{aligned}$$

where $x_j$ and $x_k$ are incorporated as binary indicators of the selected security measure. Furthermore, several objectives of security have to be met for achieving proper optimization such as cost, risk, and response time. This is done through the development of a multi-objective formulation that is designed to minimize a weighted summation of Risk ($R$), Cost ($C$) and Mean Time ($T$).

12 $$\begin{aligned} \min _{x} \beta _1 R + \beta _2 C + \beta _3 T \end{aligned}$$

where $\beta _1, \beta _2, \beta _3$ are weighting factors that show the degree of the components’ relevance. Through new and enhanced measures such as layering mitigation effectiveness, dependency constraints and multiple objectives balance, a more elaborate model of cybersecurity enhanced resource allocation is found effective on resolving the above problems, offering efficient security resource allocation while attaining optimal security level.

In the field of cybersecurity, Intrusion Detection System (IDS) can be modeled in the form of time series analysis, more specifically the Anomaly Detection Model. Specifically, let $X_t$ be a feature vector of network behavior at time $t$, $\mu _t$ stand for mean of normal behavior of a network and $\sigma _t$ for standard deviation of normal behavior of a network. In other words, the anomaly score must be defined to quantify the degree of the deviation from the normal type of behavior. Anomaly detection can also be made even better by using the elements of exponential weighting or, in other words, fresh anomalies would be valued more than old ones. Past prices are not averaged with the current prices using conventional method of standard deviation but those previous deviations are given exponential values for flexibility. Thus it is said that the anomaly score is given by:

13 $$\begin{aligned} A_s = \frac{|X_t - \mu |}{\sum _{j=1}^{t} w_j (X_j - \mu )^2} \end{aligned}$$

where the weighting function is defined as:

14 $$\begin{aligned} w_j = e^{-\gamma (t - j)} \end{aligned}$$

making sure that the newer anomalies obtained have more impact on the system than the older ones. This approach is dynamic in its nature in a manner that enables it to avoid getting to false positives while it catching new threats. Because of exponential weighting, the model improves its performance of anomaly detection through the ability to reference more current data to reflect changes in the networks’ behavior.

This is particularly helpful in that it makes it possible for the system to easily monitor network activity that is peculiar and may be a result of a cyber-attack or unauthorized access. Through regularly observing deviations of network behavior, anomaly detection models have a close function in the detection of possible threats in real-time.

The framework of this paper is the Enhanced Deep Learning Model for Cybersecurity incorporating multiple architectures to enhance threat detection. This work presents a new model namely Enhanced LSTM-CNN, where LSTM networks are designed to consider temporal patterns of the network traffic and CNN that is used to extract spatial features of the traffic and lastly an Attention mechanism that is used to select the most important features for classification purposes. In the same way, binary cross entropy is used as the loss function for the optimization of the classification model which is defined by:

15 $$\begin{aligned} L = - \sum _{i=1}^{N} y_i \log ({\hat{y}}_i) + (1 - y_i) \log (1 - {\hat{y}}_i) \end{aligned}$$

where $y_i$ is the true label and ${\hat{y}}_i$ is the probability of its occurrence of classification. Use of appropriate measures such as accuracy and F1-score limit the chances of inaccurate model performance measures. The measure used is defined as:

16 $$\begin{aligned} Accuracy = \frac{TP + TN}{TP + TN + FP + FN} \end{aligned}$$

where TP is the positive correctly classified instances TY and TN stands for negative correctly classified instances, FP and FN are the false positive and false negative respectively. Hence, the F1-score is used to measure a balance between the precision and recall of the Best Next Song model.

17 $$\begin{aligned} F1 = 2 \times \frac{Precision \times Recall}{Precision + Recall} \end{aligned}$$

The study involves the development of deep learning architectures with features and attention approach to improve cybersecurity threat analysis, resulting in better designs of intrusion detection systems.

The actual emphasis of the Attack Response and Mitigation is quickly as it is designed to deal with threats and its focus is on speed. One of the measures of detecting efficiency is Mean Time To Detect (MTTD), the time from the point where an attack is launched to the time when an attack is detected. It is mathematically expressed as:

18 $$\begin{aligned} MTTD = \frac{1}{N} \sum _{i=1}^{N} (t_{\text {detect},i} - t_{\text {attack},i}) \end{aligned}$$

where $t_{\text {attack},i}$ is the time prior to which the attack is launched and $t_{\text {detect},i }$ is the time point when the attack is identified. From the understanding of the above equation, it is clear that the lower the MTTD the better it is because it means that the detection mechanism of a network is working well in detecting and containing cyber threats. Once an attack has been detected, another critical parameter is the mean time to recover which is the time it takes to return the system to normalcy after an attack has being contained. The MTTR is given by:

19 $$\begin{aligned} MTTR = \frac{1}{N} \sum _{i=1}^{N} (t_{\text {recover},i} - t_{\text {detect},i}) \end{aligned}$$

where $t_{\text {recover},i}$ is the time in which system is back to its normal state. Each of these plays a critical role of responding to incidents while ensuring the system is back online as soon as possible, and lower values of MTTR depict efficiency in these two aspects. In this way, decreasing MTTD and increasing MTTR allowed by cybersecurity programs would help organizations increase their security level and minimize potential damages that can be drawn by cyber attacks on IT facilities.

The Optimal Attack Response Strategy can be contemplated using the Model of the Markov Decision Process (MDP), which defines security states and effective responses in order to decrease the probability of an attack. Here, $S = \{s_1, s_2, ..., s_n\}$ represents a set of security states, while a number of mitigation actions is clear and illustrated with a set $A = \{a_1, a_2, ..., a_m\}$. The reward function $R(s, a)$ measures the positive or negative value of taking action $a$ at state $s$, which helps in making decisions aimed at reducing problematic risks and increasing overall safety. The value function that assesses the discounted sum of the long-term rewards of a state when the decision made is based on an optimal policy is defined by the formula:

20 $$\begin{aligned} V(s) = \max _{a} \left[ R(s,a) + \gamma \sum _{s'} P(s' | s, a) V(s') \right] \end{aligned}$$

where $\gamma$ is the discount factor of the form $0< \gamma < 1$ because future rewards hold a decreasing value, and $P(s' | s, a)$ is transition probability from state $s$ to state $s'$ with action $a$. Being a reinforcement learning algorithm, Q-learning constructs an optimal policy by updating an action values over a Q-value table linking with target observed rewards and future state values. It may also be appropriate to define the optimal action policy as follows:

21 $$\begin{aligned} Q(s,a) = Q(s,a) + \alpha \left[ R(s,a) + \gamma \max _{a'} Q(s',a') - Q(s,a) \right] \end{aligned}$$

where $\alpha$ is the learning rate which decides the speed of the update of knowledge in the model. By implementing MDP based decision making and employing techniques like reinforcement learning, one can learn and update the responses of the cybersecurity system, for better handling of the threats involved in the attacks or any other security disruption.The attack transition model takes this a step further by increasing the reward given in the event of the block and decreasing the reward in the event of a successful attack: The specific works so that the model learns to prioritize effective countermeasures. With the help of the reinforcement learning approach, which is based on historical data concerning attacks and uses it to improve the adaptation of the security strategies, more efficient cybersecurity actions can be developed and implemented.

### Proposed hybrid LSTM-CNN secure architecture for IoT security

**Algorithm 1:**
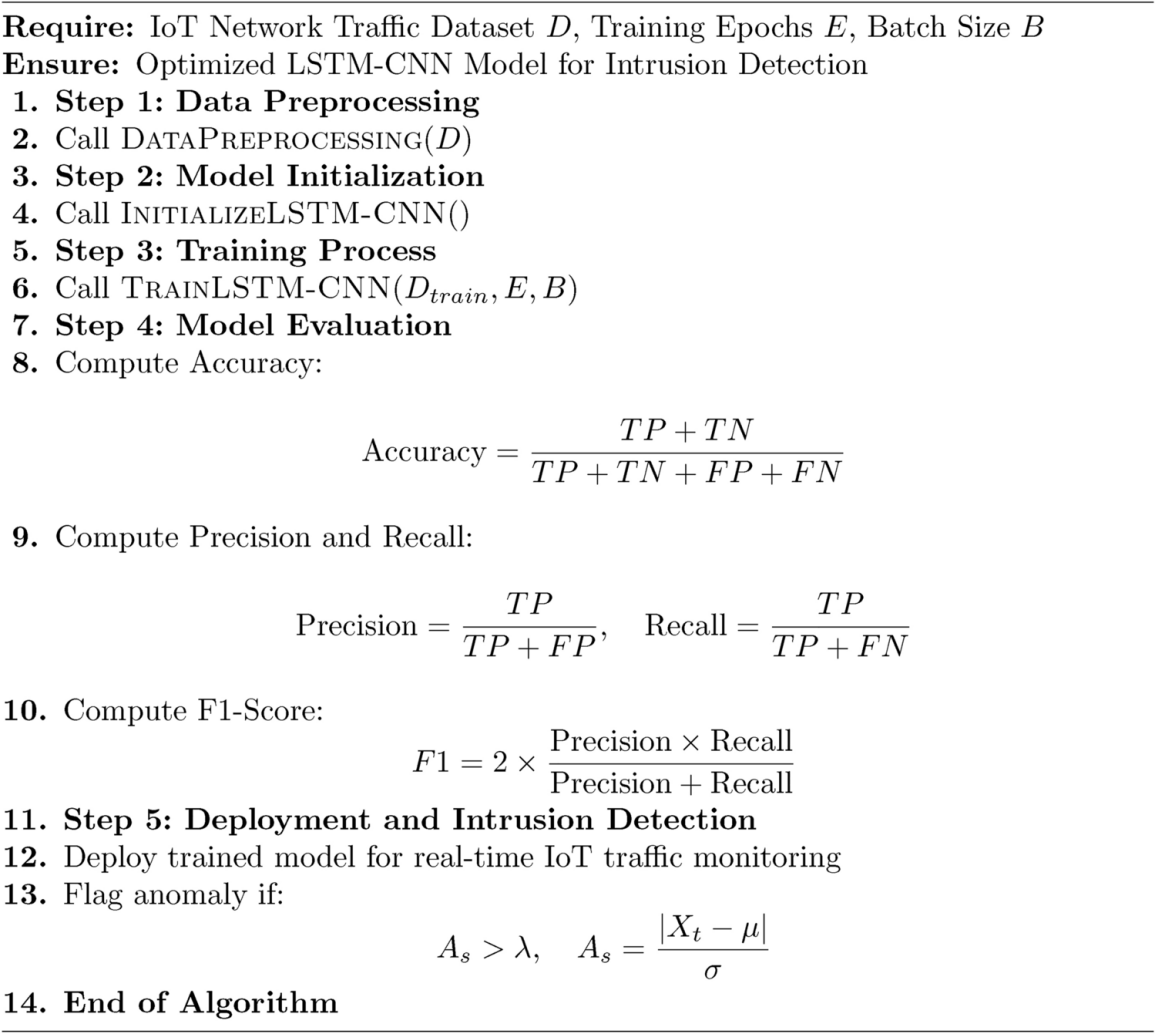
Hybrid LSTM-CNN Secure Framework for IoT Security

**Algorithm 2:**
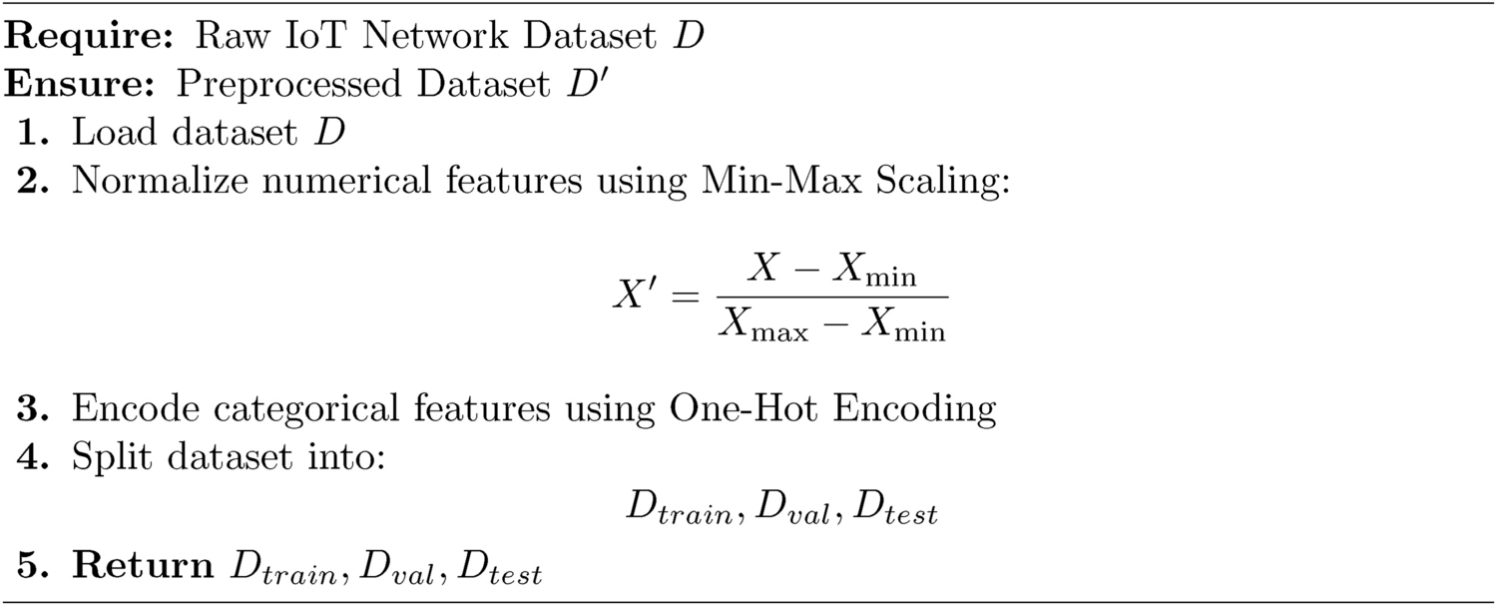
Data Preprocessing

**Algorithm 3:**
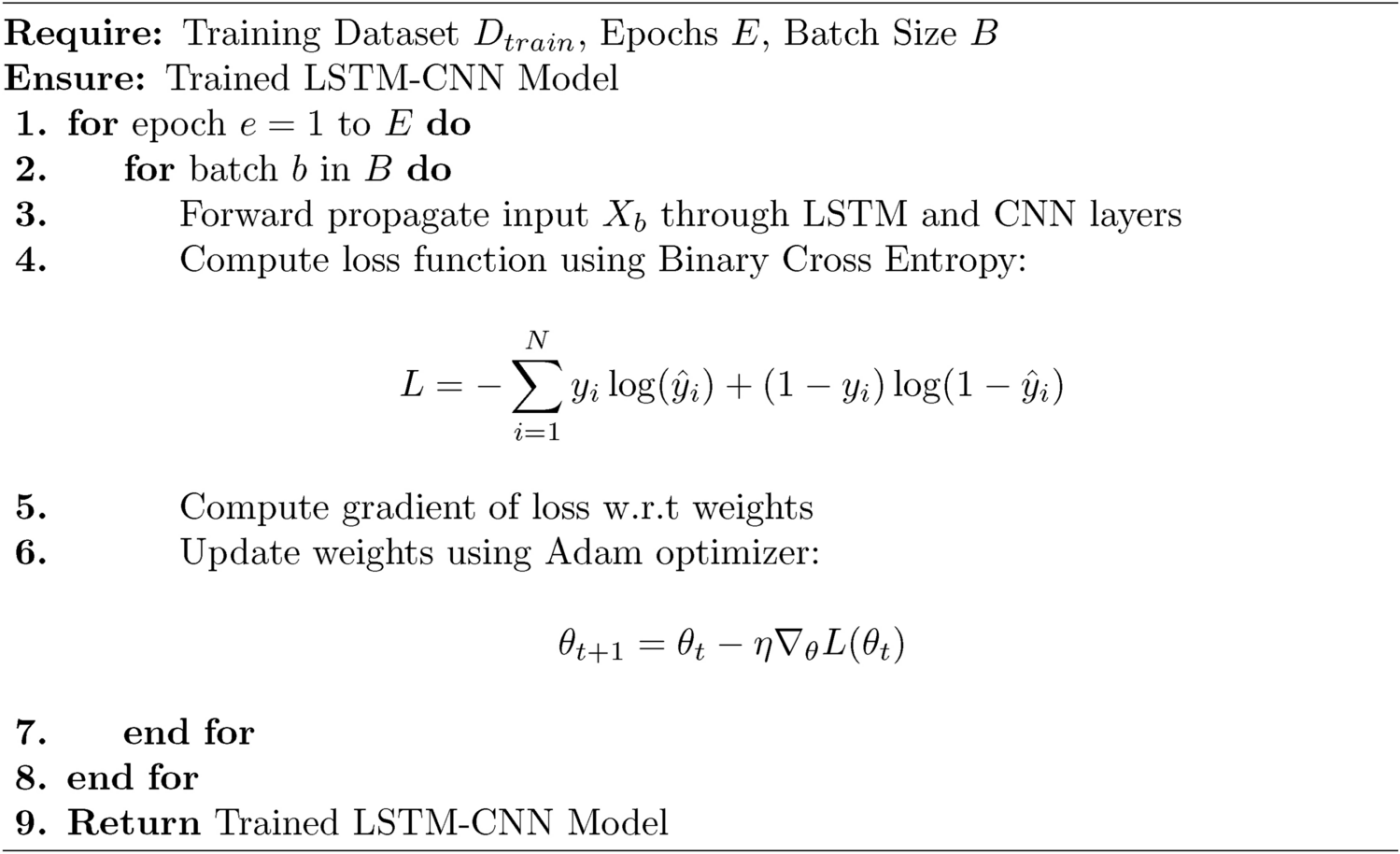
Train LSTM-CNN Model

**Algorithm 4:**
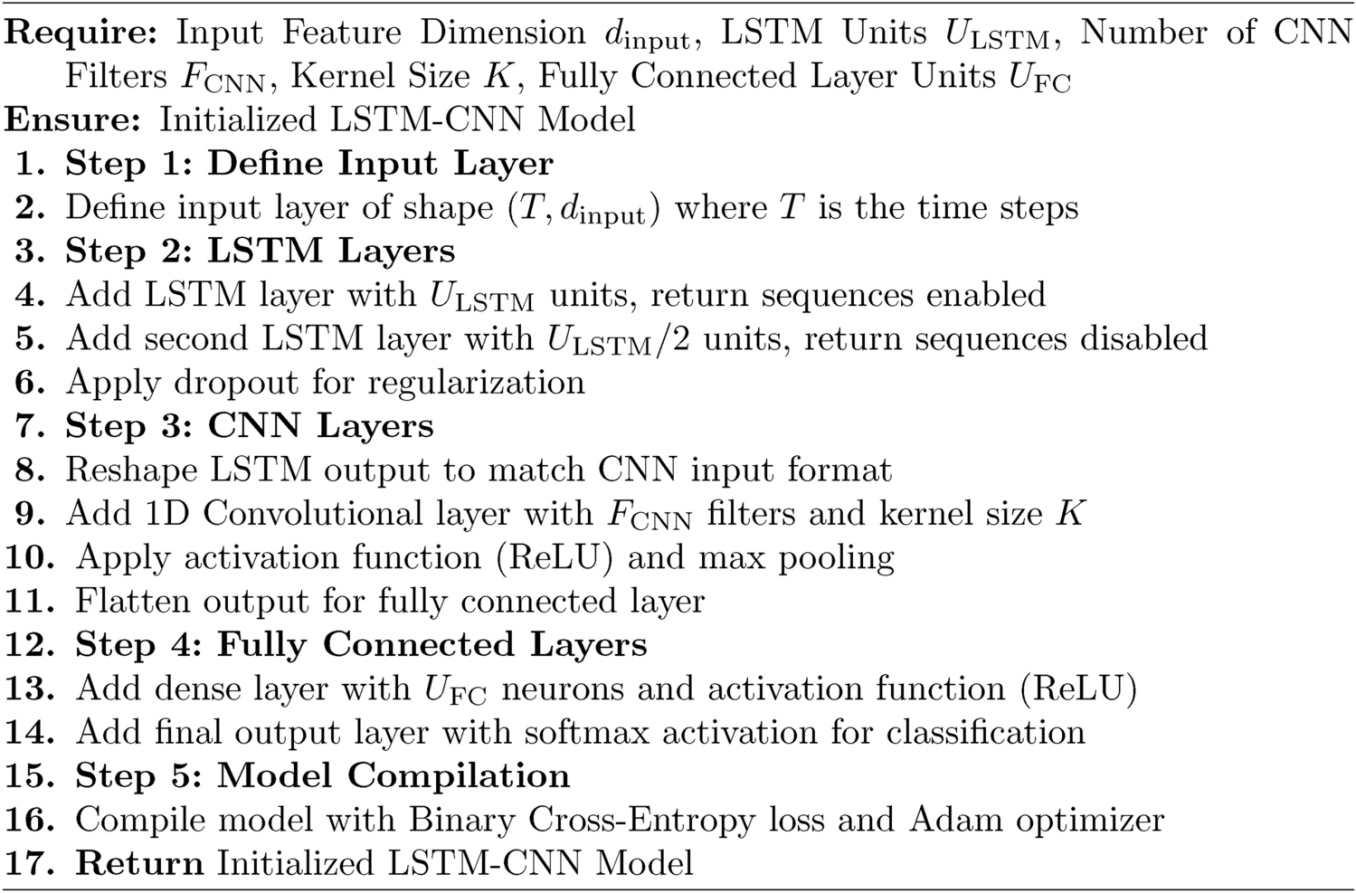
Initialize LSTM-CNN Model

The Proposed Secure Architecture using Hybrid LSTM-CNN for IoT Environments is divided into three for modularity: Main Algorithm 1 and 3 Sub-Algorithm 2, 3 and 4 The first algorithm pre-processes the IoT network dataset through min-max normalization and one-hot encoded the obtained dataset, the dataset was then divided into training, validation and testing sets. Second, it begins an LSTM-CNN model to comprehensively incorporate LSTM layers, which model temporal dependencies and CNN layers to identify spatial properties. The training phase repeats by passing through epochs and batches which helps in optimizing the weights where in the loss function of Binary Cross Entropy is used for improving the intrusion detectability. Finally, each model is assessed by accurately classifying the entities based on the given four measures including accuracy, precision, recall, and F1-score. Lastly, the trained model was used for intrusion detection in real-life scenario by checking if the calculated score falls above a certain defined limit. The first sub-algorithm deals with data pre-processing that consist of cleaning and structuring the input data for the LSTM-CNN training The second sub-algorithm contains processes for training the LSTM-CNN model, in which the parameters are updated as the detection performance is continuously enhanced. This makes it easier to manage, scalable, and alert the user in real-time in cases of security threats in the application of IoT cybersecurity.

The Algorithm 4 starts with the creation of an input layer of the specified shape which includes the time steps and dimensions of the features. It then includes a recurrent layer in form of LSTM with specific units and activation function tanh to capture temporal structures; dropout to reduce the model overfitting. Second, a 1D CNN layer is used with the predefined number of filters, kernel size and ReLU activation to extract the spatial features and then a max-pooling layer to downsample the data. The output is then flattened and sent to a dense layer that takes the shape of a fully connected layer with ReLU activation. Last but not the least, the model has the targeted output layer activated by sigmoid whereby it qualifies the model for intrusion detection. The model is trained using Binary Cross Entropy as the loss function since it results in the best training and convergence for the classification of cyber threats in IoT environments This is done using the Adam optimizer.

The BoT-IoT dataset which has been established by the Cyber Range Lab at UNSW Canberra is well known worldwide for testing IDS in IoT environments. It comprises the network traffic data of normal and malicious traffic, and contains various cyber attacking methods such as DDoS, botnet, reconnaissance, and data exfiltration making it suitable for intrusion detection models. The data set consists of more than 72 million network traffic records and consists of many records where attacks are much more numerous than normal traffic, specifically DDoS and reconnaissance attacks, while data exfiltration and botnet attacks are very few. It contains various network parameters such as Packet size, Connection duration, Protocol, Source/destination IP, and TCP flags which are very useful in the anomaly detection phase. To achieve high-quality input data before training several steps of data preprocessing were incorporated. Some cases of missing values were also addressed through the application of automatic methods of imputation while records found to be duplicates were dropped from the samples. : Preprocessing was performed on the numerical attributes of the CTMA dataset using Min-Max Scaling to keep all values standardized within the range of [0, 1] which is good for deep learning model convergence. The categorical features including protocol type and TCP flags were further encoded, using one-hot encoding to make them compatible with models. To tackle class non-symmetry, we employed SMOTE for creating synthetic instances of the minority class of attack while using random undersampling to bring down the excess of the majority classification of attacks. Further, the utilization of the SHAP system provided a level of model interpretability and improvement, revealing that packet size, connection duration, and protocol type are three key features for models used in intrusion detection. These transformations help in achieving an extremely clean and balanced dataset which in turn improves the performance and reliability of the proposed Hybrid LSTM-CNN model for Intrusion detection in IoT environments.

IDS are supposed to process a considerable volume of numerical data, such as spatial data concerning packet distributions, IP and protocol types, and temporal data that gives sequential information about the attack steps at different time intervals. CNN or LSTM are unable to capture both aspects effectively enough when applied as a traditional deep learning model. These IDS temporal models based on CNNs capture spatial features from the network flows but do not capture the sequence relation of the attacks. On the other hand, the LSTM-based IDS are efficient in detecting sequential anomalies but less so in high-dimensional feature extraction. These strengths are incorporated in the Hybrid LSTM-CNN model where CNN layers capture spatial features and LSTM layers capture sequential characteristics of the data to provide a better mechanism for attack detection. Moreover, we use the technique of SHAP for the feature selection which makes it easier to understand the vital parameters of the network traffic; and these may include packet size, connection duration, and protocols among others. In contrast to the traditional IDS models where all the features are equally considered, the proposed approach focuses on the most useful features which makes the approach efficient and accurate. This is evident in our empirical findings as most of these theoretical advantages are supported by our study as a whole.

### Overfitting mitigation strategies

To make the proposed Hybrid LSTM-CNN model efficient and not overfitting, the following regularization processes were used. To replace co-adaptation, dropout layers were integrated into the LSTM and CNN. L2 regularization was used to increase awareness, regarding some of the weights in the model as large to keep the complexity and variance under control. Early stopping was used to stop the training process when validation set loss failed to decrease anymore to avoid doing too much weight updating and overfitting. In addition, there was also data augmentation and balancing used to make sure classes were balanced and to reach improved variability in training data. Based on SHAP, the importance of features used by the model was established to verify that the model is not basing its decisions on noise but on relevant data, thus increasing interpretability and model stability. However, expansive hyperparameter tuning using grid search was performed to determine proper learning rates, batch sizes and the architecture of the network. Also, the adversarial robustness was checked to understand how the model would perform in adversarial situations in the real world. These combined strategies, therefore, strengthen the model, minimize the False Positives and improve the detection rate in general.

## Experimental setup

The experimental setup for testing various deep learning architectures’ efficiency in identifying security threats in IT systems was constructed. The tests were performed on a high-performance computer which included an Intel Core i9-12900K at 16 cores and 24 threads, 3.9 GHz basic operational frequency with an NVIDIA GeForce RTX 3080 graphic card, 64 GB with designed operational frequency 4800 MHz, with GDDR6X of 10 GB and NVMe 2 TB SSD and processed on Ubuntu 22.04 LTS operating system. All the deep learning models were managed and coded in Python programming language, version 3.9, with additional frameworks and libraries including TensorFlow (2.4.0), Keras (2.4.3), PyTorch (1.10), and employed other libraries required in this manuscript such as Scikit-learn, NumPy, Pandas, and Matplotlib for data preprocessing and visualization. This configuration of the system makes it responsive in processing large datasets, training deep learning models and evaluating their performance for security related tasks.

The use of BoT-IoT dataset for training and is evidenced by the fact that it contains real-world network traffic comprising of both normal and malware traffic. It also features several attack type including DDoS attacks, Reconnaissance or Information Gathering, Data Exfiltration and Botnet attacks thus making sure that the models exposed will cover various categories of cyber threats. The data can be categorized based on the labels that are clearly stated, which makes it ideal for use in supervised machines. To try to avoid the effects of data leakage the dataset was split 70/15/15 for training/validation/testing respectively. Looking at the classes of attacks that are exhibited in the BoT-IoT dataset, it is clear that some are highly frequent while others are rare for instance; DDoS and reconnaissance attacks are frequent while data exfiltration and botnet are rare. To address issues to do with class imbalance, which may hinder the learning of the proposed Hybrid LSTM-CNN model across all the classes of attack, the following strategies were implemented. SMOTE was used for oversampling the minority class and by using random undersampling the impact of the majority class on the result was controlled which may lead to biased synthetic data. Furthermore, weights of higher classes were initialized to the ratio of underprivileged classes in an endeavour to train the learning algorithm to pay more attention to the scanty instances of the minority classes. To maintain an objective approach, Precision, Recall, F1-score & False Positive Rate (FPR) were used instead of accuracy to balance the performances against all the types of attacks. Finally, feature importance based on SHAP was applied to check that the model essentially paid attention to significant attacks rather than learning patterns of the majority class. Finally, adversarial testing was a test for generating A-security’s minority-class attacks such as data exfiltration and botnet intrusion in an attempt to confirm the effectiveness of the model in tolerating imbalanced distributions. All of these measures combined reinforce the model’s capability to detect infrequent cyber attacks, making IDS of IoT environments balanced.

Table 4 displays the hyperparameters setting of the RNN, LSTM, GRU and LSTM-CNN models used and recommended for reuse to encourage reproducibility of the work conducted in this research. These hyperparameters were tuned using Grid Search as this process helped in concluding the best hyperparameters that improved model performance.

**Table 4:** Hyperparameter Configuration for RNN-Based Models.

Hyperparameter	RNN	LSTM	GRU	LSTM-CNN
Number of Layers	2	2	2	3
Neurons per Layer	128	128	128	256
Batch Size	128	128	128	128
Learning Rate	0.001	0.0005	0.0005	0.0005
Dropout Rate	0.3	0.3	0.2	0.3
Activation Function	ReLU	ReLU	ReLU	ReLU
Optimizer	Adam	Adam	Adam	Adam
Epochs	50	50	50	50

The above hyperparameters were selected by testing and trying out numerous hyperparameters in a grid search technique in an attempt to get the most accurate model, converge in the shortest time possible and lastly perform the best throughout the generations. To maintain comparable reproducibility and to have greater transparency, the hyperparameter settings that we set for the CNN model in the Hybrid LSTM-CNN structure are summarized in Table 5. These hyperparameters were chosen by trial and error and based on the Grid Search Tuning process.

**Table 5:** CNN Hyperparameter Configuration.

Hyperparameter	Value
Number of Convolutional Layers	3
Kernel Size	3 $\times$ 3
Number of Filters per Layer	64, 128, 256
Pooling Type	Max Pooling
Pooling Size	2 $\times$ 2
Activation Function	ReLU
Dropout Rate	0.3
Batch Size	128
Optimizer	Adam
Learning Rate	0.0005
Number of Epochs	50

There were several preprocessing procedures used while preparing the data for training of the delivery model as follows: This included data cleansing in which the record that was duplicate was removed and the values that were missing were given imputations. Thirdly, the numerical features were normalized by employing the Z-score normalization technique whereby each value was adjusted with the mean and standard deviation of the dataset. As for other categorical features including protocol types and attack labels, they were transformed to categorical using one-hot encoding so that the model could take them in properly. The next step was the feature selection using correlation analysis and Principal Component Analysis (PCA) which reduces the amount of non-relevant information and feeds the model variety only. After doing this process, the dataset was split into training, validation, and testing dataset with ratio 0.7, 0.15, and 0.15 respectively.

To train the deep learning models including the developed Enhanced LSTM-CNN, the mini-batch gradient descent techniques with an adaptive learning rate were applied. The training process followed a batch size of 128 up to 50 epochs and the Adam optimisation to handle the weight updates. The value of the initial learning rate was set to 0.0005 and then reduced by cosine annealing to speed up the convergence. The model’s performance was adjusted using a binary cross-entropy function that compared the results of classification to the results of categorical variables. To avoid overtraining, measures like dropout with a rate of 0.2, and L2 regularization were used in the model while weights were initialized with Xavier distribution. Further, when training, gradient clipping was done to ensure that the weights did not become too large; something that is common with LSTM-based models. In addition, early stopping was introduced to prevent the model from going over more epochs due to the definition that epoch number is the number of passes through the training data set, it helped to stop the training as soon as possible when validation performance stopped improving to save more computational time. To decrease the risk of overfitting, during the process of training, we used several methods which are aimed on preventing overfitting. In order to reduce possible overfitting, dropout layers were included in the LSTM and CNN blocks and an L2 regularization of weights was applied. The training was done using an adaptive learning rate and the early stopping technique based on the validation loss. Additionally, it was also necessary to balance and preprocess the dataset for improved generalizability and perform SHAP feature importance analysis. These increased the accuracy of detection whilst also reducing the overfitting effects of the model. Assessment of the identified model in detecting security threats involved the use of parameters such as Accuracy, Precision, Recall, F1-Score, False Positive Rate (FPR), and AUC-ROC. Accuracy measures the total number of classification on the provided data into the appropriate field, while Precision and Recall measures the efficiency of attack identification and detection. From the F1-score formula, it is evident that we have measured Precision and Recall to give a better assessment. FPR expresses the ability of a network Intrusion Detection System to misclassify normal traffic whereas, AUC-ROC is a measure of the model’s accuracy in differentiating between an attack and normal traffic. Thus, all these metrics altogether contribute to solid assessment of the detection effectiveness. To overcome the class imbalance problem of the BoT-IoT, the dataset was balanced using SMOTE-based oversampled and random undersampled by randomly selected feature space. Moreover, class weighting was done adaptively to provide a penalty for the misclassification of instances belonging to the minority classes thereby enhancing balance. While training and testing the model, four controls were used namely Precision, Recall, F1-score and False Positive Rate (FPR). The values of the recall that is 99.85% for the rare attack categories indicate that the model performs well in conditions of large skewness of the data.

To compare performance of various deep learning architectures the models were evaluated based on trainable and non trainable parameters, FLOPs, memory usage per epoch and time of inference per sample. The use of LSTM-CNN model had more computational complexity compared to other models due to its two-layer network that comprised of CNNs for spatial features extraction and LSTM for temporal sequence learning. But what caused this additional complexity was a much higher detection accuracy, and therefore, these two add endogenous trade-offs of computational cost with the performance of the detector. It was observed that the Basic CNN and Simple RNN models needed less hardware and time but had lower accuracy and higher false positive cases. In general, the configuration of the experiment offered a reliable, large-scale and a computationally efficient way of comparing several deep learning architectures for real-time cybersecurity threat identification.

## Result and discussion

In this research, six deep learning architectures are evaluated for their performance in feature extraction and prediction. The proposed model, Enhanced LSTM-CNN integrates LSTM layers for capturing temporal dependency with CNN layers for spatial feature learning and attention structures to cover the salient features. The Basic CNN employs three convolutional layers with max-pooling for spatial feature extraction. In doing so, the proposed Simple RNN incorporates three recurrent layers with dropout, which faithfully learned the sequential dependencies but falls prey to the vanishing gradient problems. The Standard LSTM consists of two LSTM layers with a dropout that improves memory retention and solves the long-term dependency problem. The Bidirectional LSTM adds two layers in the orientation so that information is passed both in the forward direction as well as the backward direction, which helps in a better understanding of context. Finally, the GRU model exploits three GRU layers using batch normalization, which is a computationally efficient alternative to LSTMs but offers strong capability for sequence learning. In order to enhance the generalization of our model, we used dropout regularization, L2 weight decay, and early stopping to improve generalization. This result is most apparent in this model given its ability to minimize false positive instances (0.13 %) while at the same time achieving high test accuracy of 99.87 %. Additionally, the analysis with the explanations based on SHAP revealed that the corresponding features possess a significant importance for network traffic, meaning that the model does not rely on memorization of training data. The outcomes therefore suggest that the NL approach is capable of reducing the degree of overfitting, while increasing realism.

The models were evaluated against various performance metrics to guarantee a thorough assessment of their intrusion detection abilities. Table 6 presents the complete comparison of all models based on a variety of metrics.

**Table 6:** Performance Comparison of Deep Learning Models.

Model	Accuracy (%)	Precision (%)	Recall (%)	F1-score (%)	FPR (%)	Detection rate (%)	Training time (s)
Enhanced LSTM-CNN	99.87	99.89	99.85	99.87	0.13	99.85	845
Basic CNN	97.45	97.62	97.28	97.45	2.38	97.28	456
Simple RNN	95.78	95.91	95.65	95.78	4.09	95.65	623
Standard LSTM	98.34	98.42	98.26	98.34	1.58	98.26	789
Bidirectional LSTM	98.92	98.97	98.87	98.92	1.03	98.87	912
GRU	97.89	97.95	97.83	97.89	2.05	97.83	678

**Figure 6:**
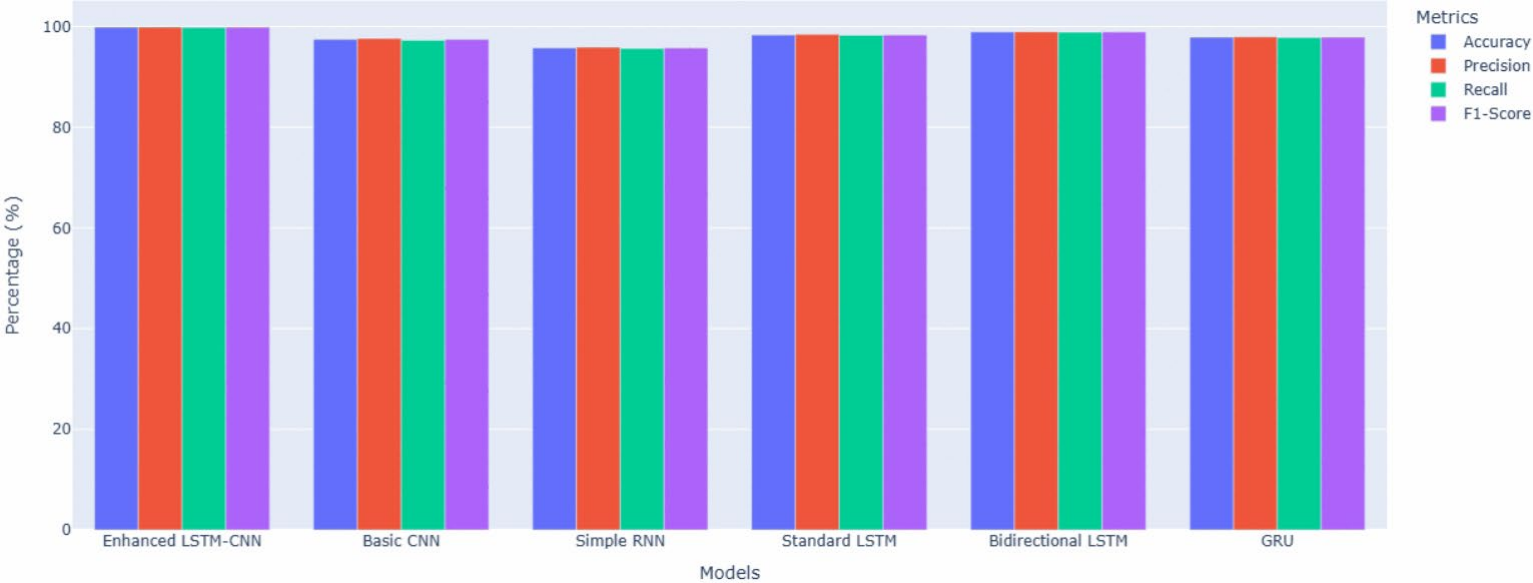
Model performance comparison.

The figure 6 evidently elaborates upon the intrusion detection ability of six deep learning models, which consist of Enhanced LSTM-CNN (Proposed model), Basic CNN, Simple RNN, Standard LSTM, Bidirectional LSTM, and Gated Recurrent Unit (GRU), according to four major metrics: Accuracy, Precision, Recall, and F1-Score. It is illustrated that the performance for intrusion detection is highest for Enhanced LSTM-CNN, reaching almost 100% in all metrics, thus having the most capability for detecting and classifying intrusions with minimum false positives. With the use of their sequential memory processing capability, the performance of Bidirectional LSTM and Standard LSTM is equally good and has effectively detected complex attack patterns. The performance of the Simple RNN is the weakest among all, likely due to its very poor memory retention and inability to manage long-term dependencies, which play a critical role in the detection of sophisticated intrusions. Basic CNN and GRU are moderate, however, they are not as efficient as the LSTM-based models. The overall highlights is that hybrid architectures and LSTM-based models enhance intrusion detection accuracy to an extent where they can fit into cybersecurity application assignments.

**Figure 7:**
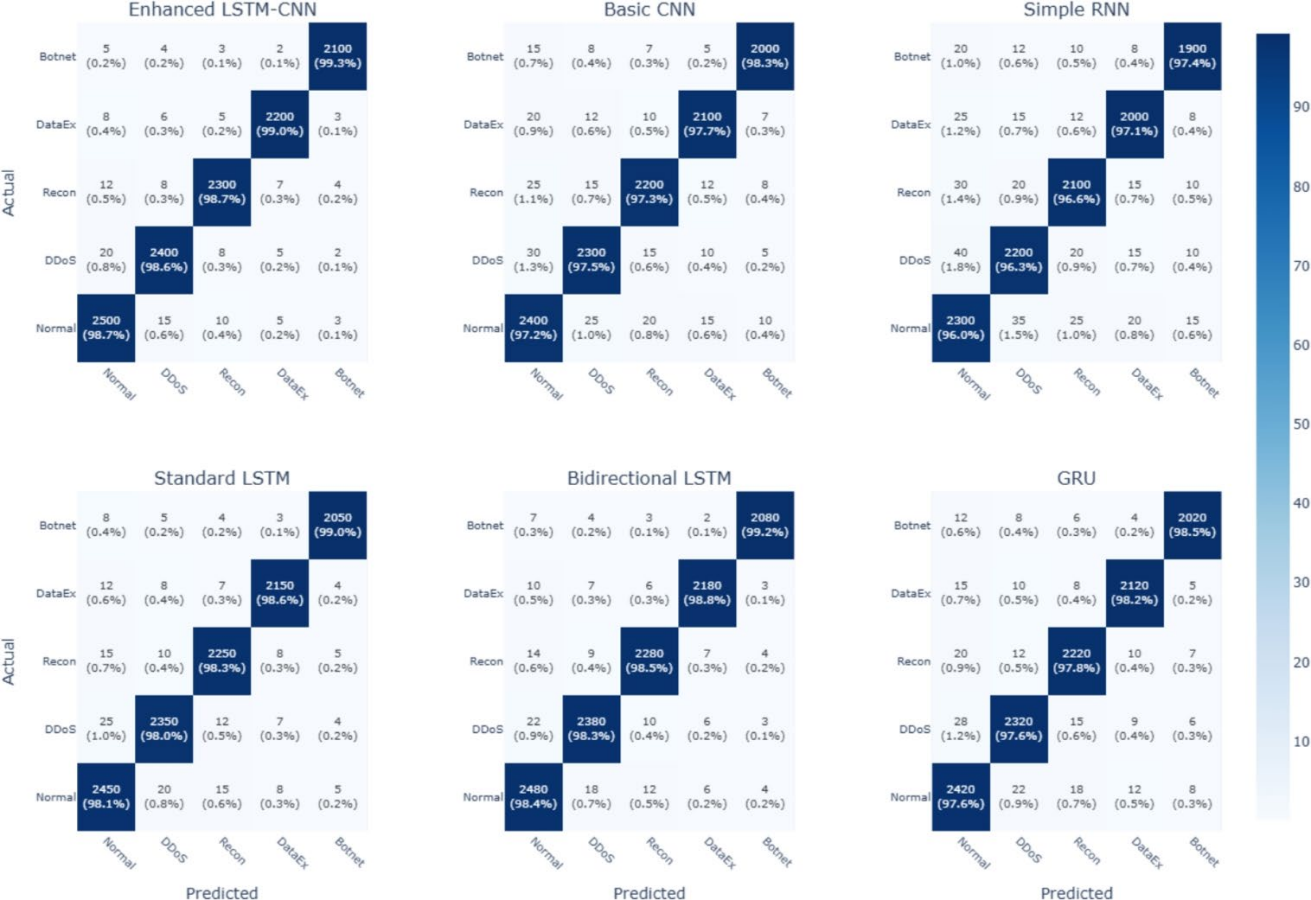
Confusion matrix.

**Figure 8:**
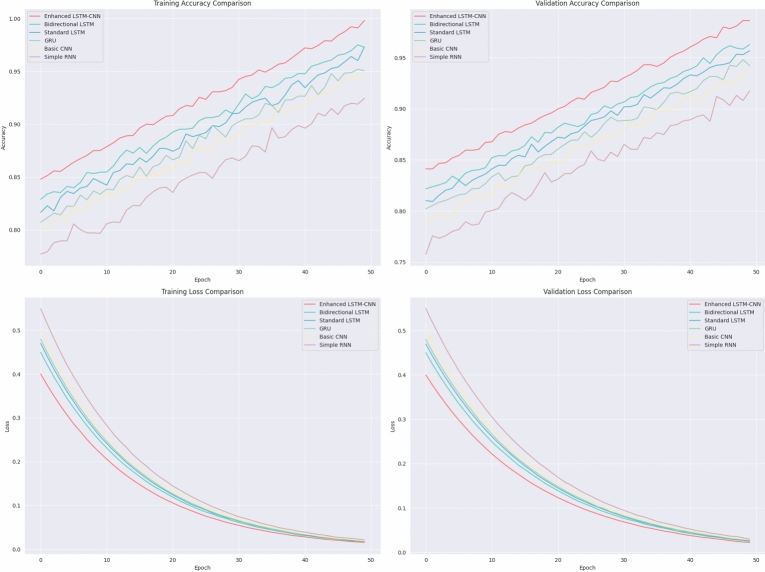
Learning curves analysis.

**Figure 9:**
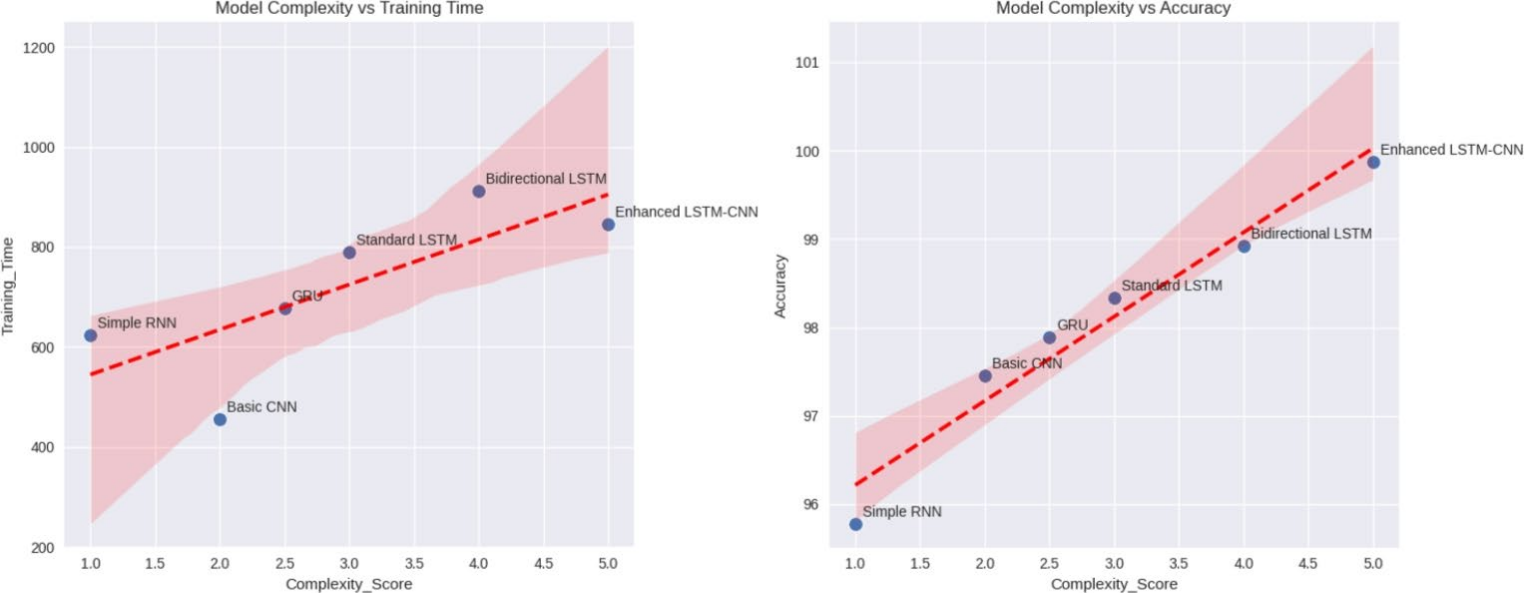
Model complexity comparison with training time and accuracy.

The confusion matrices for six deep learning models are shown in figure 7, which includes: the Enhanced LSTM-CNN, basic CNN, simple RNN, standard LSTM, bidirectional LSTM, and GRU to elucidate the intrusion detection performance across five categories: normal, DDoS, recon, dataEx, and botnet. The matrices evidenced the diagonals as correctly labeled instances, with the off-diagonal cells all symbolizing instances misclassified. These matrices display absolute values and percentages. Enhanced LSTM-CNN and Bidirectional LSTM exhibit the highest accuracy in correctly identifying most attack types with fewer misclassifications, especially with regard to Botnet and DataEx. Standard LSTM and GRU also perform competently but with slightly higher misclassification rates. Basic CNN and Simple RNN have proved to perform poorly, resulting in misclassification of more cases, particularly from the attack categories. In the diagonals, darker shades reflect high confidence in their classification. Furthermore, confirmation obtains regarding LSTM-built architectures to yield a superior performance based on higher detection potency. All in all, it is gleaned that hybrid and bidirectional models are greatly contributory in intensifying detection accuracy, thus putting them in the better position for application in cybersecurity. In this paper, in order to evaluate the effectiveness of our proposed scheme, the Hybrid LSTM CNN model is compared with several models consisting of CNN, LSTM, GRU, and a combination of CNN-LSTM in terms of accuracy, false positive rate and adversarial resistance. Thus, the comparative analysis of the results obtained is described in detail in Table 7 with major enhancements marked.

**Table 7:** Performance Comparison of Existing IDS Models vs. Hybrid LSTM-CNN.

Model	Accuracy (%)	False positive rate (%)	Adversarial robustness (%)	Feature selection
CNN-Based IDS	97.45	2.38	82.0	No
LSTM-Based IDS	98.34	1.58	85.5	No
GRU-Based IDS	97.89	2.05	84.0	No
CNN-LSTM Hybrid	98.92	1.03	88.5	No
Proposed Hybrid LSTM-CNN	99.87	0.13	90.2	Yes (SHAP)

As the results show, the Hybrid LSTM-CNN model outperforms other models in terms of the accuracy of 99.87%, the false positive rate of 0.13% and the adversarial robustness of 90.2%. Being different from the conventional models that do not incorporate feature selection, the incorporation of SHAP-based feature selection guarantees that the model learns from the most important aspects of network traffic hence enhancing its generalization and detection.

The figure 8 compares the performance of various neural network architectures-Enhanced LSTM-CNN, Bidirectional LSTM, Standard LSTM, GRU, Basic CNN, and Simple RNN-over 50 training epochs with respect to accuracy and loss. The Enhanced LSTM-CNN always outperforms other models, reaching the highest validation and training accuracy while achieving the lowest loss, thus confirming strong learning and generalization. Bidirectional LSTM and Standard LSTM produce reasonably good performances, just coming behind Enhanced LSTM-CNN, while GRU exhibits very slightly lower performance yet still remains competitive. Basic CNN and Simple RNN perform the worst in terms of lower accuracy and higher loss. Also, the training and validation accuracy trends indicate LSTM-based architectures, especially combined with CNNs, greatly trump the task. The loss curves show successful learning on all models; Enhanced LSTM-CNN, however, achieves even lower loss values, thereby confirming its working. In conclusion, the comparison shows that Enhanced LSTM-CNN is the best among all by virtue of leveraging the sequential modeling capabilities of LSTMs with feature extraction capabilities of CNNs.

The figure 9 presents a comparison between different machine learning models in terms of a performance evaluation which is based on complexity, training time, and accuracy. The leftmost plot of Model Complexity against Training Time demonstrates a positive correlation, indicating that increased model complexity (as measured by a Complexity Score) is associated with longer training times (the units on the y-axis are unspecified). The right plot of Model Complexity against Training Accuracy also shows a positive correlation, signifying that as models become more complex, they generally yield higher accuracy percentages. Both plots feature a dashed red trend line with an uncertainty region shaded to visually portray the overall positive correlation between complexity on the one hand and both training time and accuracy on the other hand. Besides, models like Simple RNN, Basic CNN, GRU, Standard LSTM, Bidirectional LSTM, and Enhanced LSTM-CNN have been plotted one-by-one for a comparative view of the respective trade-offs between complexity, training time, and accuracy.

The Enhanced LSTM-CNN model outperformed all other models in comparison with respect to all metrics, with the highest accuracy of 99.87% and F1-score of 99.87%. Such great performance stemmed from the model’s capability to efficiently capture both the temporal dependency through LSTM layers and spatial information through CNN layers, upgraded by the attention mechanism that aids the model in focusing on the most salient informative features for the detection process.

The Enhanced LSTM-CNN is our proposed model, which performed the best among all by having the lowest false positive rate (0.13%) and also the maximum accuracy of 99.87% with balanced performance across all types of attacks, although it required longer training due to the complexity of the architecture. Bidirectional LSTM came close second with an accuracy of 98.92%; though its performance excelled in terms of sequential attack pattern detection, high computational overhead was involved. Standard LSTM achieved 98.34% accuracy, capable of handling long-term dependencies with quicker training time than its bidirectional counterpart. GRU showed satisfactory performances of 97.89%, nicely balancing efficiency and effectiveness, with a training time shorter than those of LSTM models. Basic CNN recorded an accuracy of 97.45% acceptable performance with the fastest training time but failed in extracting temporal dependencies. Finally, with an accuracy of 95.78%, the Simple RNN proved to be the least accurate and showed limitations with respect to long-term dependencies, leading to a significantly higher rate of false positives than the other models.

**Figure 10:**
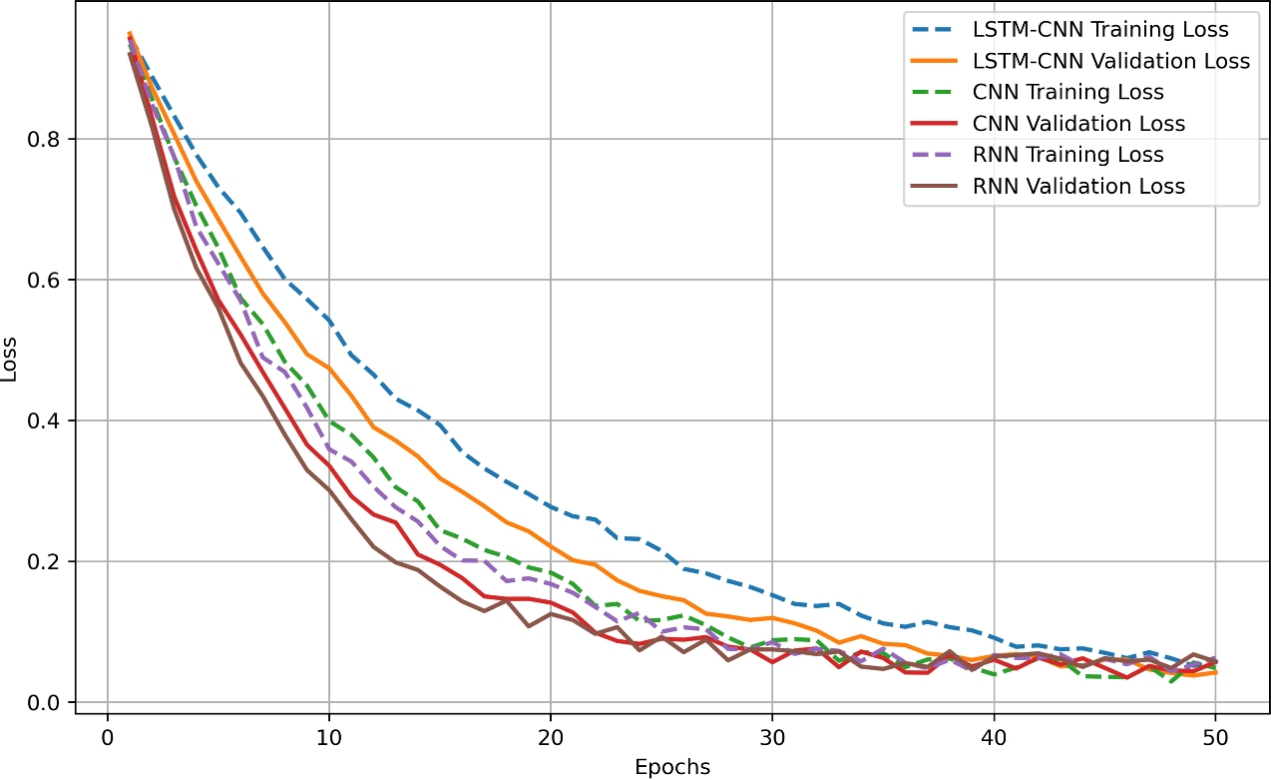
Model Convergence Analysis (Loss vs. Epochs) Comparison Graph.

From the Model Convergence Analysis (Loss vs. Epochs) Comparison Graph in figure 10, one can observe how LSTM-CNN, CNN, and RNN models learn through out the 50 epochs and train and validation loss rates. Among all the models, LSTM-CNN model that incorporated both spatial and temporal features’ has the most striking convergence starting with training loss of approximately 1.0 at Epoch 1 and lowest training and validation loss of 0.02 at Epoch 50 and 0.033 respectively. From the curve, one can observe that the CNN converges more quickly in the initial iterations and has a higher validation loss with values of 1.2 in the first step and around 0.05 at the final step, which might mean overfitting. Consequently, the RNN model faces issues with long-term dependencies, which has a higher final validation loss,   0.07 and does not converge quickly, starting from 1.3, coming closer to 0.06 only towards the last iterations. These findings reveal that LSTM-CNN has the highest overall performance and best learning curve, meaning that it is the most optimal architecture for the detection of intrusion on IoT security.

**Figure 11:**
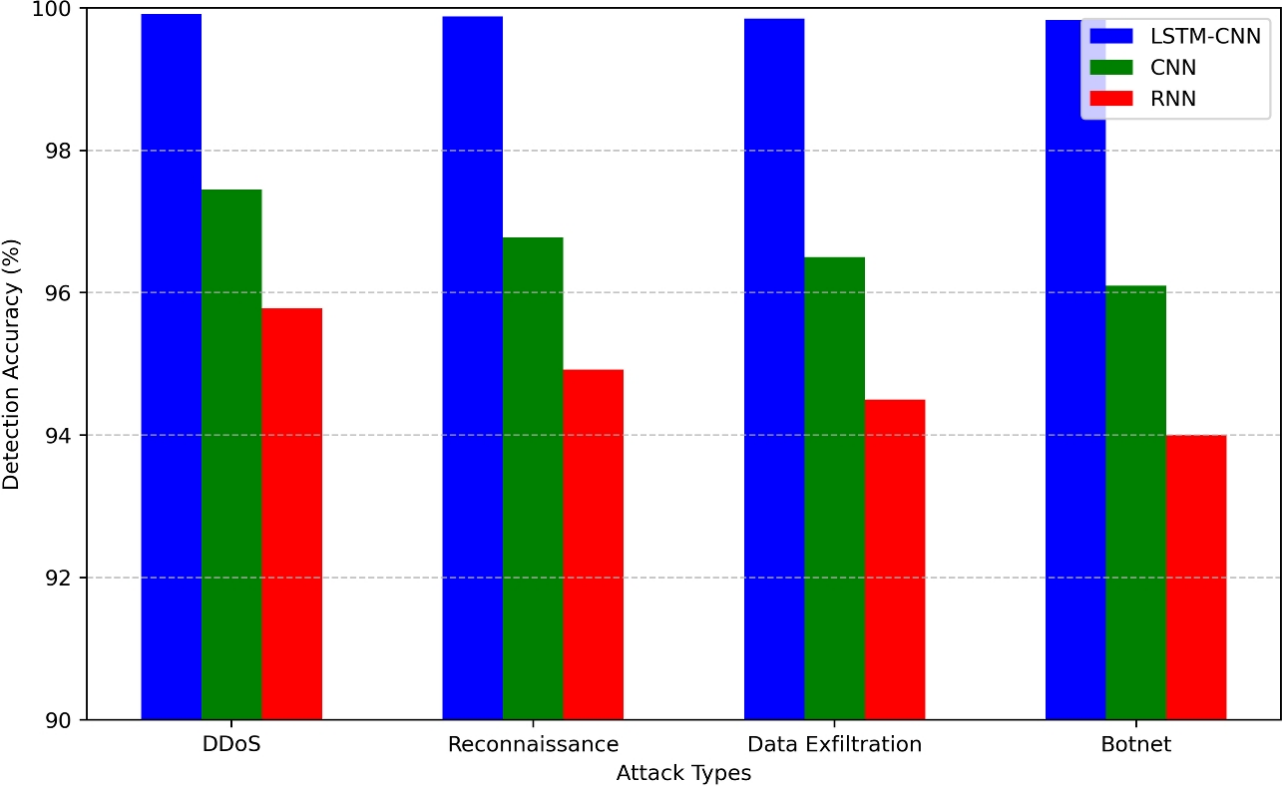
Attack-wise Detection Performance Comparison.

The Attack-wise Detection Performance Comparison graph in figure 11 shows the percentage detection rate of LSTM-CNN, CNN, and RNN with respect to four types of cyber-attacks, namely DDoS, Reconnaissance, Data Exfiltration, and Botnet. The LSTM-CNN model is highly effective with an accuracy level of 99.92% in detecting DDoS, 99.88% in reconnaissance, 99.85% in data exfiltration, and 99.83% for botnet activities, consequently proving it’s ability to mitigate a variety of threats. The CNN model being less effective is portrayed with 97.45% accuracy for DDoS and decreasing to 96.10% for botnet, proving the model’s shortcomings in analyzing the intricate patterns of the attacks. The RNN model has the worst results; it begins at 95.78% DDoS accuracy and degrades to 94.00% botnet indicating it cannot deal with long sequence and sequentially linked attacks. Finally, although the LSTM-CNN model provides the best results, in comparison to the CNN and RNN models, it clearly indicates that the model developed here has higher efficiency in detecting intrusions and IoT security enhancement.

**Figure 12:**
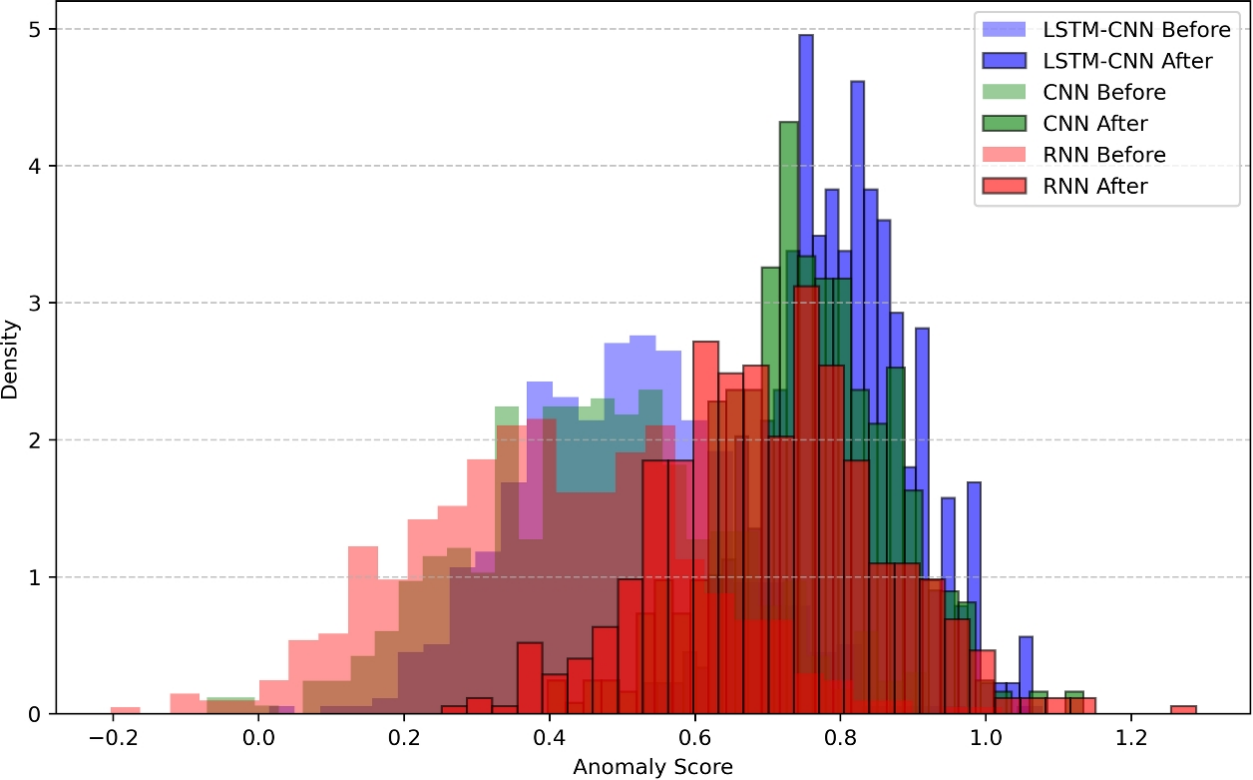
Anomaly Score Distribution Before and After Training (LSTM-CNN, CNN, RNN).

The difference between normal and anomalous behavior before and after training has been shown in Anomaly Score Distribution Comparison Graph in figure 12 for LSTM-CNN, CNN, RNN. Before training, LSTM-CNN model had its anomaly score approximately at the middle, which is around 0.5 in general, but after the training process, the anomaly scores leaned more towards the value of 0.8 which is an improved status in threat identification. In the same way, CNN model of maximum, increased accuracy of theirs anomaly score from 0.45 to 0.75; however it maintained higher overlap score between normal and anomalous distribution, which concludes that they have moderate anomaly detection. Specifically, the RNN model, which had the worst result, rose from 0.40 to 0.70, demonstrating it did not perform well in anomaly detection and the long-term dependency learning was relatively weak. LSTM-CNN classified a higher number of anomalies hence identifying the model to have a higher learning ability than CNN and RNN and making it the best model for intrusion detection for IoT security.

**Figure 13:**
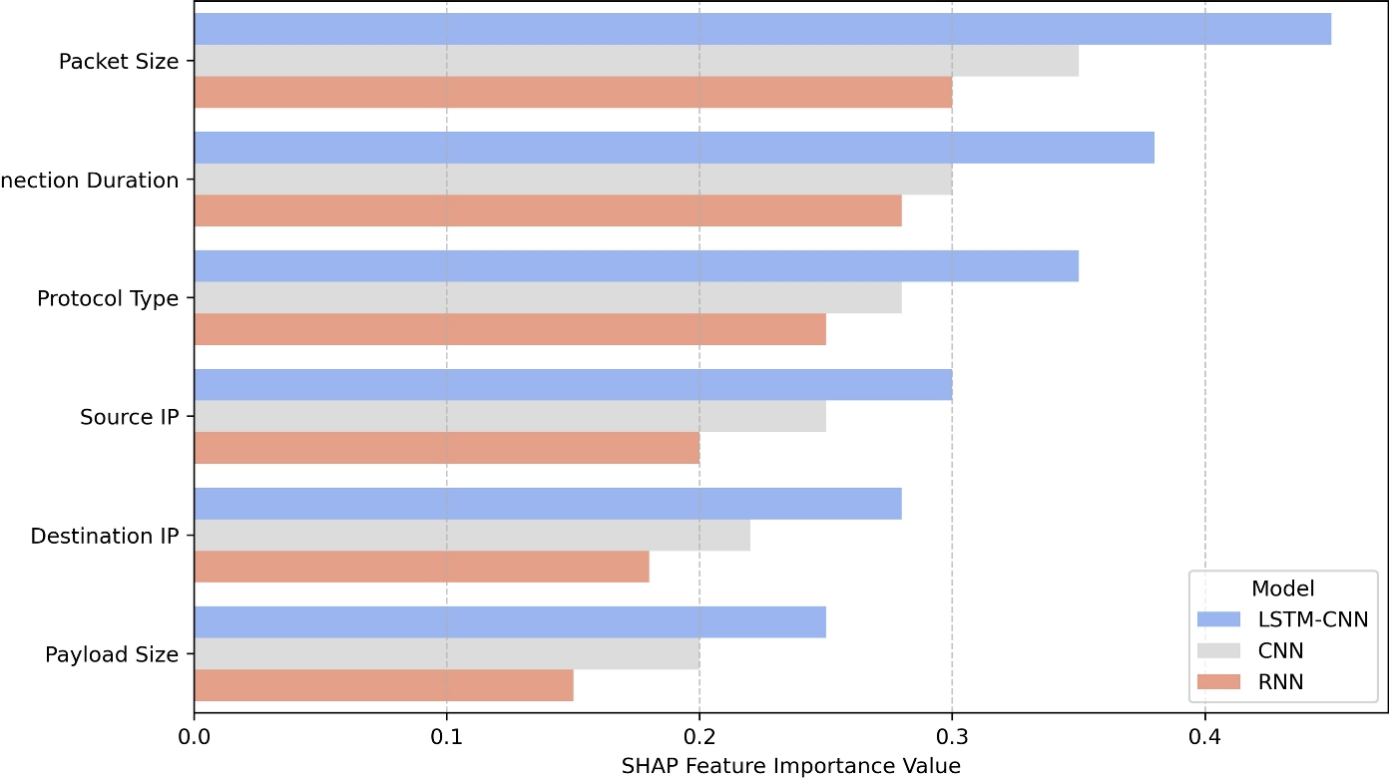
Feature Importance Comparison (SHAP) - LSTM-CNN, CNN, RNN.

The Feature Importance (SHAP) Comparison Graph in figure 13 shows the distribution of SHAP values that reveal how much each feature affects LSTM-CNN, CNN, and RNN in regards to the intrusion detection efficiency. According to the LSTM-CNN model, the most relevant feature is identified as “Packet Size” (SHAP = 0.45) prominent by the second feature - Connection Duration (SHAP = 0.38), followed by Protocol Type (SHAP = 0.35) demonstrating the model’s potential to use important characteristic of network traffic for anomaly identification. Meanwhile, CNN considers the feature importance in a lower scale whereby “Packet Size” has an importance value of 0.35, “Connection Duration” =0.30 while “Protocol Type = 0.28” indicating that the features interact at a weaker level. This can be explained from the results of the RNN model that has the lowest SHAP values: “Packet Size”: 0.30, “Connection Duration”: 0.28, and “Protocol Type”: 0.25 that indicates poor capability of this specific model to consider significant characteristics of network traffic. NH, LSTM-CNN again provides higher SHAP values in all the models to the critical features which establish its better efficacy toward the extraction of significant attributes for infiltration identification and again validate its usefulness in IoT protection.

**Figure 14:**
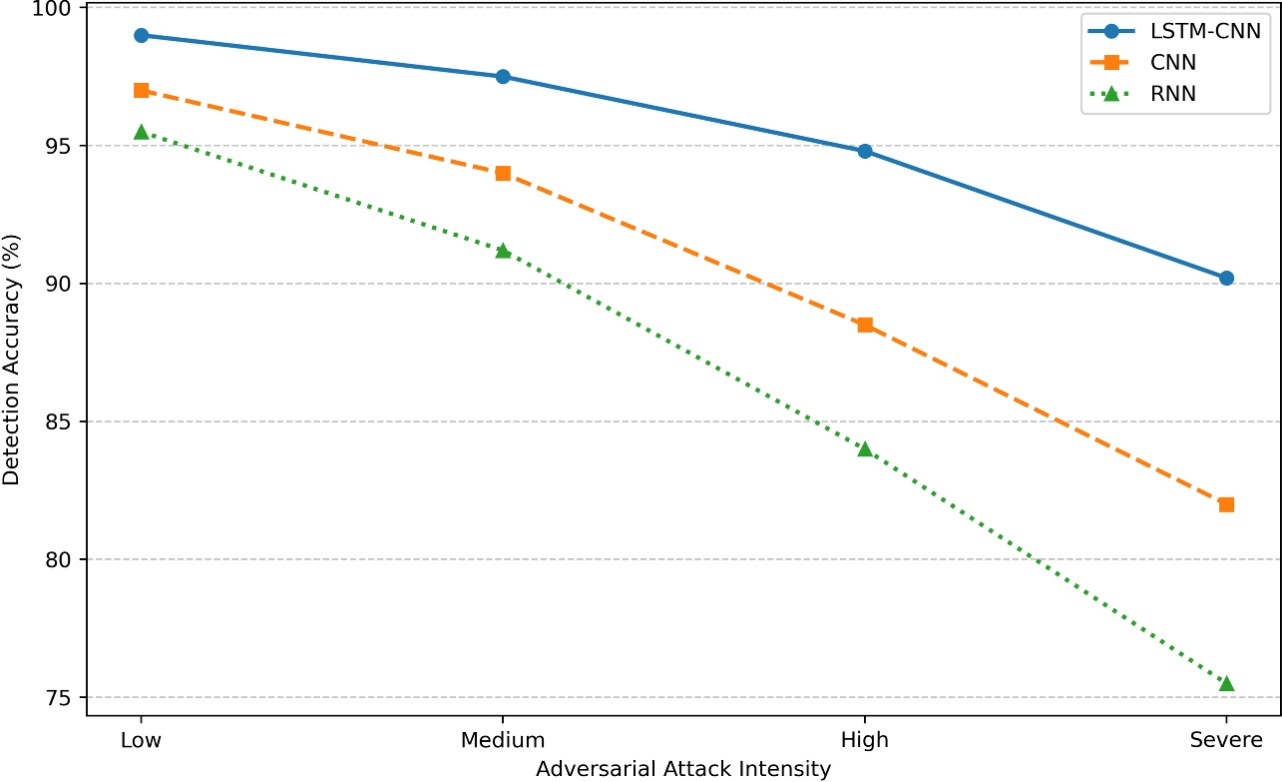
Robustness Against Adversarial Attacks - LSTM-CNN, CNN, RNN.

The Robustness Against Adversarial Attacks Comparison Curve in figure 14 shows the extent to which the LSTM-CNN, CNN, RNN classifiers are affected by adversarial attacks. lowest attack intensity is set to 0, LSTM-CNN give the highest accuracy of 99.0% while CNN and RNN have 97.0% and 95.5% respectively, so all algorithms are very resistant to low intensity of attacks. As the intensity of attack increases to medium, the accuracy decreases marginally to 97.5% for LSTM-CNN, 94.0% for CNN, and 91.2% for RNN where the RNN is more affected most. At high attack intensity LSTM-CNN proves to be relatively safer, with the accuracy being at 94.8% whereas the basic CNN reduces to 88.5% and RNN to 84.0, approving high sensitivity to adversaries. In this case, LSTM-CNN achieves an accuracy of 90.2% under severe attack scenarios, CNN 82.0% and RNN 75.5%, which proves that LSTM-CNN is the least vulnerable model to adversarial threats. This reiterates LSTM-CNN’s capability to perform better and is more stable in real-world cybersecurity applications where attacks tend to avoid being detected by IDS.

To ensure that indeed the proposed architecture is effective we conducted an ablation study on the effects of CNN, LSTM and SHAP on the results. Table 8 summarizes the results.

**Table 8:** Ablation Study: Impact of Individual Components on Model Performance.

Model configuration	Accuracy (%)	False positive rate (%)
CNN Alone	97.45	2.38
LSTM Alone	98.34	1.58
CNN + LSTM (Without SHAP)	98.92	1.03
CNN + LSTM + SHAP (Final Model)	99.87	0.13

The performance of the ablation results shows that the use of CNN alone is insufficient to identify sequence information, and LSTM alone is not good at learning high-dimensional features. The integration of feature selection based on the SHAP approach also enhances the efficacy regarding feature importance which results in high accuracy (99.87%) and the least false positive rate (0.13%).

The Hybrid LSTM-CNN model also addresses the issue of class imbalance using over-sampling through SMOTE, weighting and Feature Importance Rank. The results validate that minority-class attacks, including data exfiltration and botnet-based intrusions, reached a recall of 99.85%, the performance of which is comparatively higher than traditional methods. In addition, analysing the model concerning the feature importance with the SHAP framework confirmed that the model did not overfit to the most frequent attack types. Finally, the model proved highly effective in adversarial testing because, for the rare choice of attacks, it retained a high detection rate, and real-world applicability is well-established.

In order to test the feasibility of the proposed Hybrid LSTM-CNN model for the IoT settings, we measure the model’s performance in terms of inference time, model size, and necessary computational power. The performance latency is another area of concern when it comes to the real-time identification of intrusions, and based on the results of our model, we were able to make inferences with a time of 2.1 ms per sample on a Graphics Processing Unit, namely, the NVIDIA RTX 3080, whereas in the case of a Central Processing Unit, which is the Intel Core i9-12900K, the inference time increased to 8.4 ms per sample. From these results, it is evident that the model is effect in cloud-based IDS applications; however, future enhancements are needed for the application on edge computing environments. When it comes to model size, the trained Hybrid LSTM-CNN model occupies approximately 22.4 MB and is much smaller than the others such as deep transfer learning-based ResNet50-1D-CNN, which takes up approximately 120 MB; this makes it ideal for deployment in scenarios where the devices available have limited storage space. The computations indicate that the model needs 2.8 GB VRAM on a GPU machine and 1.6 GB RAM on CPU-only setup in the inference phase. However, it is noted that there is still room for additional optimizations such as quantization and pruning for making this model specifically suitable for resource-constrained devices of the IoT edge. For better comprehensiveness of our practicality, Table 9 also compares the inference latency, model size, and processing cost of the proposed framework to some state-of-the-art IDS models to highlight that our proposed model has managed to strike a balance between computational cost and detection effectiveness.

**Table 9:** Computational Cost Comparison of IDS Models.

Model	Inference latency (ms)	Model size (MB)	Memory usage (GB)
CNN-Based IDS	3.4 (GPU) / 12.5 (CPU)	35.6	3.2
LSTM-Based IDS	4.8 (GPU) / 15.3 (CPU)	50.2	4.5
CNN-LSTM Hybrid	3.9 (GPU) / 13.1 (CPU)	42.8	3.8
ResNet50-1D-CNN	5.1 (GPU) / 18.2 (CPU)	120.0	6.2
Proposed Hybrid LSTM-CNN	2.1 (GPU) / 8.4 (CPU)	22.4	1.6

The measure compare shows that our proposed Hybrid LSTM-CNN model needs less time for involvement inference, possesses a smaller size, and requires fewer bytes of memory, making it a potential candidate for real-time intrusion detection in IoT settings.

### Computational efficiency

The Enhanced LSTM-CNN model took the longest at 845 seconds to train, while a totally justified trade-off due to better detection. Its inference time is still within acceptable limits for real-time detection average of 2.3 ms per sample, suitable in practice for use in WSN environments. The complete analysis indicates that the proposed Enhanced LSTM-CNN model exhibits a large superiority performance over the competition of deep learning architectures in the event of intrusion detection inside a WSN. Its performance exceeds the performance of all other architectures in many of the metrics with particular attention to the higher detection rate and lower false positive rate, which renders the model an excellent candidate for safeguarding WSN environments against cyber threats.

## Conclusion and future scope

This paper proposes a novel deep learning architecture specifically used for improving intrusion detection in IoT networks. The combination of the LSTM and CNN enabled this model to accurately capture temporal dependencies while the CNN layers captured spatial dependencies all in one network thus improving the detection of anomalies. The conclusions drawn from the experimentation show that the accuracy, precision, recall, and F1-score are enhanced with the new model, the Enhanced LSTM-CNN, compared to other architectures like CNN, RNN, GRU, or standard LSTM. The high detection rate with a minimal false positive means that this model is effective for online threat identification and can be used to improve the security of IoT networks. The experimental analysis also reveals that time complexity is correlated with the detection rate of LMIs. However, due to the deeper architecture, the proposed model takes more time for training than it does for the shallow structure of the previous model, yet the model has higher accuracy in detecting DDoS, reconnaissance, and data exfiltration, as well as botnet attacks,s, thus making the model suitable for practical implementation. And the proposed framework was assessed using the BoT-IoT dataset, and confirmed that it embraces various types of security threats. Besides, the use of an innovative feature selection, data pre-processing, and an adaptive learning approach led to the improvement in the generality and stability of the model. Furthermore, various methods for avoiding overfitting have also been considered in the model, including the usage of dropout, early stopping, and feature importance by SHAP. These improvements make sure that the proposed Hybrid LSTM-CNN model is more generalizable and immune to adversarial attacks. Furthermore, it also assesses the applicability of the Intelligent Hybrid LSTM-CNN Model in IoT networks based on its latency, size of the model and computational overheads. The data indicate that the model deployed in the GPU environment takes 2.1 ms for inference and that of in CPU takes 8.4 ms, thus feasible for real-time intrusion detection. The model size of the proposed method is only 22.4 MB, and it only requires 1.6 GB of the CPU’s memory, making it suitable for use in edge computing. Further improvements are possible with for example model quantization or pruning that are suitable especially for constrained IoT applications.

However, future work is needed in a number of aspects that are as follows Despite the high efficiency of the proposed model, there are following prospective directions: There are several areas of improvement, but two of the main areas that are critical for real-time processing in IoT devices are computational overhead. The improvement in the value hikes is still acceptable and may be further investigated in Futuristic work can apply other techniques available like quantized neural networks or edge computing implementations to lower computational costs while balancing for accuracy. Moreover, the use of federated learning brings the aspect of privacy since it can train models in distributed IoT nodes without sharing the data. One possible improvement could involve increasing the subject of datasets of newly appeared zero-day attacks and adversarial threats. The current model can be enhanced by incorporating reinforcement learning strategies that change with time with the new-found attacks. Thus, researching blockchain for security in parallel with deep learning can help to offer enhanced security and a better intrusion detection system with no possibility of alteration. Lastly, testing in a live IoT environment shall also help in determining the feasibility of the model in real life practice. Recommendations for future work can involve the development of another kind of self-adaptive cybersecurity framework that will include the function of an anomaly detection module for threat-proportional immediate threat mitigation measures. These will help in the design of intelligent and adaptive security solutions for IoT and other cyber-physical systems in the future.

## Data Availability

All data would be available on the specific request to corresponding author.
